# Childhood Material Hardship Linked to Adolescent Neurocognition: A Computational Modeling Approach

**DOI:** 10.1111/desc.70213

**Published:** 2026-05-05

**Authors:** Yue Linda Zhang, Alexander S. Weigard, Felicia A. Hardi, Sunghyun H. Hong, Edward Huntley, Colter Mitchell, Luke W. Hyde, Christopher S. Monk

**Affiliations:** ^1^ Department of Psychology University of Michigan Ann Arbor Michigan USA; ^2^ Department of Psychiatry University of Michigan Ann Arbor Michigan USA; ^3^ Wu Tsai Institute & Department of Psychology Yale University New Haven Connecticut USA; ^4^ School of Social Work University of Michigan Ann Arbor Michigan USA; ^5^ Survey Research Center of the Institute for Social Research University of Michigan Ann Arbor Michigan USA; ^6^ Population Studies Center of the Institute for Social Research University of Michigan Ann Arbor Michigan USA; ^7^ Neuroscience Graduate Program University of Michigan Ann Arbor Michigan USA

**Keywords:** adversity, cognition, development, diffusion decision model, material hardship

## Abstract

Childhood material hardship, including insecurity in housing, utilities, food, and medical care, is a critical factor influencing cognitive functioning and mental health outcomes. However, past research on material hardship is limited by cross‐sectional data and reliance on conventional behavioral measures of cognitive functioning that suffer from poor reliability and lack of specificity. To address these gaps, this study used a well‐validated computational model of cognition to examine individuals’ drift rate, which is hypothesized to be driven by the efficiency of evidence accumulation (EEA) for decision‐relevant information, a key process that supports higher‐order cognitive functioning. Here, we examined how material hardship during childhood was associated with adolescent drift rate, and whether drift rate was linked with attention difficulties. 187 adolescents recruited from the Future of Families and Child Wellbeing Study were included in the analyses. Adolescents exposed to greater material hardship showed lower drift rate, suggesting less effective processing. Growth curve modeling revealed that initial exposure to material hardship, but not changes across childhood, was associated with drift rate in adolescence. Lower drift rate was also associated with concurrent attentional problems and served as a significant indirect pathway linking material hardship to adolescent attention. This is the first longitudinal study that examined the associations among childhood material hardship exposure, adolescent drift rate, and attentional difficulties. Our results suggest that challenges to essential living conditions in childhood may impact cognitive processes underlying goal‐directed behavior in adolescents. These findings highlight the application of computational models to reveal specific cognitive processes impacted by adversity.

**Summary:**

- Existing research on material hardship and cognitive functioning is limited by reliance on cross‐sectional task‐based performance, resulting in inconsistent findings.
- We applied the diffusion‐decision model to decompose trial‐wise performance into underlying cognitive processes, including drift rate, a key evidence accumulation process underlying goal‐directed behavior.
- Greater cumulative childhood material hardship, beyond exposure to other adversity, is associated with lower drift rate, which is linked with greater attentional problems in adolescents.
- Findings were specific to drift rate, not average response time or accuracy, emphasizing the value of computational methods in capturing cognitive changes associated with material hardship.

## 1 Introduction

Growing up in poverty poses significant challenges for the development of cognitive functioning and mental health (Lawson et al. 2018; Müller and Kerns 2015). Poverty‐related hardships can shape the development of higher‐order processes that facilitate attention, inhibition, and behavioral regulation, which has long‐lasting implications for academic achievement, mental and physical health (Diamond 2013; Feinstein and Bynner 2004; Moffitt et al. 2011). However, measures of socioeconomic disadvantage frequently fail to capture the daily challenges, such as insecure housing and lack of nutritious food, that are experienced by many families. Furthermore, methodological issues exist in how cognitive functioning is measured across studies. To address these gaps, the current study investigated how material hardship, a measure of the lived challenges of poverty, is associated with drift rate, a cognitive process measured using a well‐validated computational model of cognition that has been shown to be a key underpinning of goal‐directed behavior. Drawing on 15 years of longitudinal data from the Future of Families and Child Wellbeing Study (FFCWS), results from this study shed light on the unique impact of material hardship on adolescent cognitive development, highlighting the importance of using computational approaches to better understand the associations between socioeconomic disadvantage, cognitive functioning with implications for attentional problems in adolescents.

### 1.1 Material Hardship, Developmental Risks, and Cognitive Functioning

Nearly one in five children in the United States live in poverty, with over 40% having experienced challenges meeting basic needs (U.S. Census Bureau 2023). The widespread prevalence of poverty has prompted extensive research into its psychological consequences, revealing that growing up in poverty is linked to adverse developmental outcomes (Haushofer and Fehr 2014; Peverill et al. 2021; Schenck‐Fontaine and Ryan 2022). However, common indicators of poverty, such as socioeconomic status or income level, offer limited insight into the daily realities of those affected because the impact of family income can vary significantly depending on factors such as cost of living and social support. To better capture the lived experience of poverty, researchers have advocated for alternative measures to income or socioeconomic status. One such measure is material hardship, which provides a more direct assessment of tangible challenges, such as food insecurity, housing instability, and difficulties meeting basic needs (Gershoff et al. 2007). Notably, experiences of hardship can occur in families that have income levels above the poverty line, which remains a relatively low benchmark (e.g., $30,000 for a family of four in 2023).

Material hardship during childhood has been increasingly recognized as a critical factor influencing developmental outcomes. Exposure to such hardship in childhood and adolescence can undermine psychological well‐being, increasing the risk of anxiety, depression, and externalizing behaviors (Huang et al. 2021; Pechtel and Pizzagalli 2011; Peverill et al. 2021). A less investigated developmental outcome is attention‐related functioning. Prior studies have linked poverty and related risk factors to elevated attention difficulties in children and adolescents, though findings remain mixed and intertwined with genetic influences (de Zeeuw et al. 2020; Russell et al. 2016). Less work has specifically examined the specific role of material hardship on attentional difficulties, often combining it with factors like environmental pollution (Dellefratte et al. 2019; Perera et al. 2018). Some studies have found significant associations of food insecurity and housing tenure with attentional problems from childhood to adolescence, but findings on the strength and consistency of these effects remain mixed (Hanson 2025; Lu et al. 2019; Paquin et al. 2021; Russell et al. 2015).

While research that specifically examined the effects of material hardship on cognitive functioning remains limited, broader evidence suggests that early exposure to aspects of material hardship, such as food insecurity and housing crowding, is associated with lower cognitive skills across various domains, including math, reading, and problem‐solving (Alaimo et al. 2001; Jyoti et al. 2005; Schenck‐Fontaine and Ryan 2022; Solari and Mare 2012). Beyond material hardship, socioeconomic disadvantage has long been linked to deficits in cognitive functions such as inhibition, task‐switching, and working memory in childhood and adolescence (Hackman et al. 2015; Lawson et al. 2018). These findings align with theories suggesting that chronic stress from adverse experiences—including economic hardship—can alter neural development in regions responsible for higher‐order cognitive processes, resulting in decreases in functioning (Farah et al. 2006; Sheridan et al. 2020). Recent theoretical framework suggests that these changes are not just deficits, as adversity‐exposed youth may also develop context‐sensitive adaptations in cognition that reflect trade‐offs rather than impairments (Ellis et al. 2022). Empirical work has indeed found support that certain cognitive abilities may adapt in response to harsh environments to match the challenges of that environment (Fields et al. 2021; Mittal et al. 2015; Young et al. 2022). This complexity highlights the need for more nuanced approaches to studying cognition under adversity.

### 1.2 Measurement of Cognitive Functioning: Drift Rate and EEA

Cognitive functioning has frequently been assessed with performance on behavioral tasks, typically measured by mean response times (RTs) and accuracy/error rates (Johnson et al. 2021; Wei et al. 2025). These tasks often rely on fast and accurate responses to assess individuals’ ability in particular cognitive domains (Farah et al. 2006; Fields et al. 2021). However, this approach has several limitations. First, cognitive assessments encompass a diverse range of tasks that measures different domains of higher‐order functioning, including simple decision‐making, inhibition, and attention shifting (Diamond 2013; Nigg 2017). The variability in tasks used across studies makes direct comparisons challenging and introduces psychometric issues, such as poor test‐retest reliability of many commonly used measures (Eisenberg et al. 2019; Hedge et al. 2018; Price et al. 2015). A second issue is that traditional task performance measures are often “black box” aggregates that obscure the many cognitive processes involved in completing a task. For example, fast and accurate performance on an inhibition task is typically assumed to reflect stronger inhibitory control. However, task performance is also influenced by unrelated factors such as response caution and motor response speed (Hedge et al. 2018; Lerche and Voss 2019), which are typically not accounted for. As a result, findings from prior research may not reflect, as intended, differences in cognitive ability, but instead be confounded by other processes that are not directly related to the construct of interest.

**FIGURE 1:**
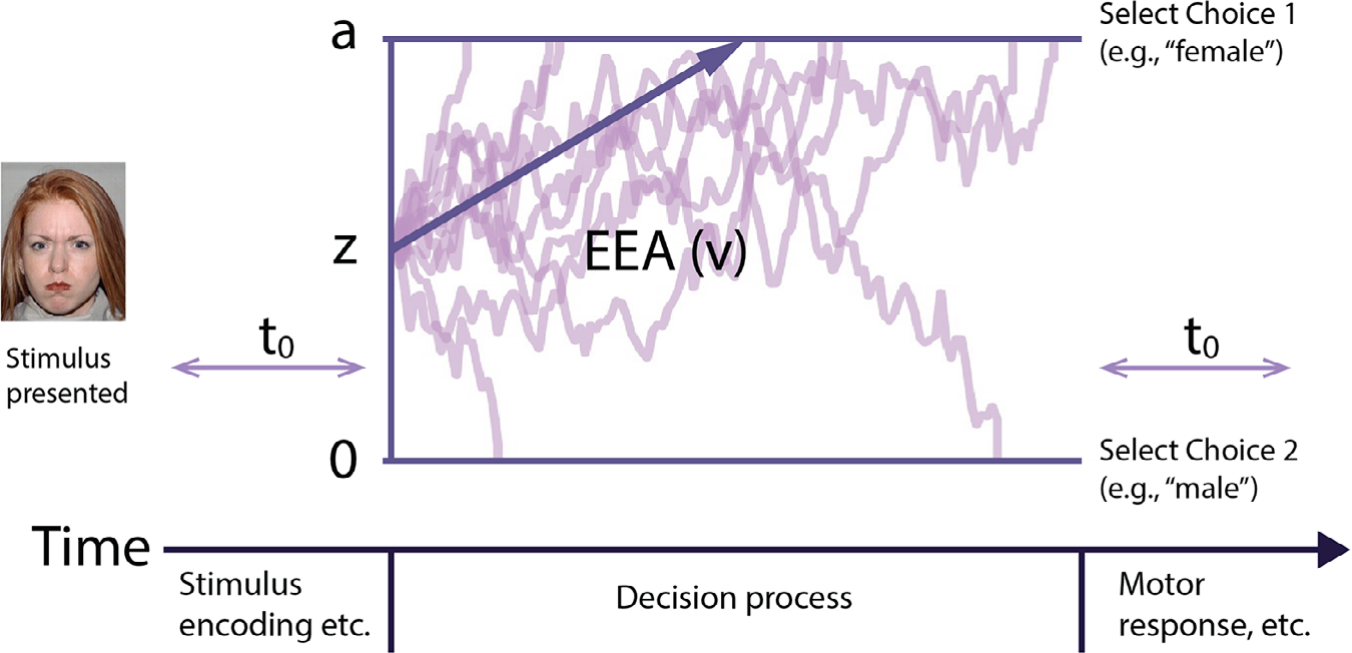
**Schematic of the diffusion decision model (DDM) in a single trial**. The model describes a forced two‐choice decision task. This example shows the emotional‐faces gender identification task used in the current study. After the stimulus has been presented, the trial is decomposed into a decision component and a non‐decision component. Within the decision component, the DDM assumes a single decision variable that represents the amount of relative evidence for one of the two possible choices (i.e., male or female in this example). The decision variable starts from a starting point (z) and drifts over time in a single trial between the two boundaries representing the two choices. Once the decision variable reaches a boundary, the corresponding choice is made. The width of separation between two choice boundaries is boundary separation (a), which represents how much evidence is needed before being able to make a choice. The rate at which evidence accumulation drifts towards the boundary is drift rate (v), or EEA. Finally, the non‐decision time (t_0_) represented the combined time spent for any task preparation (e.g., stimulus encoding) and response execution (e.g., button pressing).

A promising solution to these issues is the application of formal mathematical models that describe neurocognitive processes underlying task performance. The Diffusion Decision Model (DDM, also known as Drift Diffusion Model; Figure 1) is a well‐validated model that explains individual behavior on simple two‐choice decision tasks and has been widely used in cognitive psychology (Ratcliff and McKoon 2008; Voss et al. 2004, Voss et al. 2013). The model posits that within each trial, people gather evidence for possible responses continuously until a critical threshold has been reached for either one of the two options. Once the threshold is reached, the participant will make the decision. The model also accounts for preparation and execution processes that occur outside of decision‐making as well as an individual's level of caution in responding (i.e., prioritizing accuracy vs. speed). By integrating RT and accuracy information on a trial‐by‐trial basis, the model provides a more nuanced and reliable measure of cognitive function than traditional behavioral indices (Voss et al. 2004).

The DDM decomposes task performance into several underlying cognitive processes, each represented by distinct parameters that influence behavior (Voss et al. 2004). Four parameters from the DDM are commonly examined. The drift rate (v) reflects how efficiently an individual gathers goal‐relevant information to make a decision that is consistent with the goals of the task. Boundary separation (a) represents response caution, with larger values indicating a more conservative decision strategy that requires more information before committing to a choice. Starting point (z) reflects response bias, or the tendency to favor one option over another, such as when one response is more probable. Finally, non‐decision time (t_0_) accounts for the time it takes for processes unrelated to the decision stage, such as stimulus encoding and motor execution of response.

Among these parameters, the drift rate has received particular attention in the literature because drift rates across diverse tasks appear to be largely explained by a general trait‐like factor, termed efficiency of evidence accumulation (EEA), that drives individual differences in task performance across many domains of cognitive functioning (Weigard et al. 2021; Weigard and Sripada 2021). This task‐general process has been supported by studies using latent factor approaches, which show that drift rates from diverse tasks load moderately to strongly (typically .50–80) onto a single EEA factor (Löffler et al. 2024; Schubert et al. 2016; Vermeent et al. 2024). EEA has also demonstrated strong test‐retest reliability, internal consistency, and cross‐task validity compared to conventional behavioral measures (Eisenberg et al. 2019; Lerche et al. 2020; Schubert et al. 2016; Tomlinson et al. 2025). Although the most robust estimates of task‐general EEA are derived from performance across multiple tasks, prior research suggests that drift rate estimates from even single tasks can serve as reasonable approximations of the broader EEA construct, including based on tasks that do not have a focus on higher‐order cognitive functioning, such as simple perceptual decision‐making tasks (Löffler et al. 2024; Weigard et al. 2021). Given its conceptual links to general cognitive functioning, EEA has been increasingly used to study cognitive processes in a variety of contexts and has been implicated as a transdiagnostic trait in understanding externalizing behaviors, substance use, and depression, among other outcomes (Duyser et al. 2025; Letkiewicz et al. 2023; Paige et al. 2024; Weigard and Sripada 2021). The most extensive use of DDM in examining psychopathology has been in research on ADHD and related attentional challenges, where individuals with ADHD consistently exhibit reduced EEA across various tasks, including simple perceptual decision‐making and inhibition tasks, further supporting the idea that EEA is a task‐general cognitive trait (see Weigard and Sripada 2021 and Ziegler et al. 2016 for detailed reviews).

### 1.3 Drift Rate, Material Hardship, and Attentional Problems

Despite the implication of drift rate and EEA in understanding attention‐related symptoms and the methodological advantages of DDM, the cognitive parameter has yet to be examined as a mechanism through which material hardship can increase risk for attentional difficulties. In addition, only a few studies to date have used DDM to examine the associations between material hardship and drift rate as an index of cognitive ability. One study found that while material hardship was not significantly associated with changes in drift rate across cognitive domains (e.g., processing speed, inhibition, and attentional control), greater exposure to threat was linked with lower EEA (Vermeent et al. 2024). Additionally, two other cross‐sectional studies have explored the relationship between material hardship, among other types of adversity, and other computational parameters derived from DDM or similar modeling approaches, finding mixed results with how hardship exposure are impacting aspects of cognition (He et al. 2024; Vermeent et al. 2024). In addition to the limited research on the topic, no longitudinal studies have been conducted to examine the link between material hardship exposure, or poverty broadly, and adolescent drift rate, leaving a gap in understanding the cumulative effects of childhood material hardship on cognitive development. Longitudinal studies allow for examination of the accumulation of hardship exposure, which has been shown to have critical importance for adolescent development (Evans et al. 2013; Hardi et al. 2024). Beyond cumulative exposure, evidence also suggests that changes in poverty and hardship experience across time may be important to consider, such that decrease in household economic status is associated with decrease in cognitive outcomes (Levesque et al. 2021; McLeod and Shanahan 1996). These changes in hardship experiences over time can be captured using longitudinal assessments. Therefore, further research is needed to clarify these links in a longitudinal framework.

As discussed, most research examining task‐based cognitive performance in relation to adverse experiences and poverty has relied on RT and accuracy as primary measures with the oversimplistic assumption that slower RTs and higher error rates necessarily indicate lower cognitive functioning. The DDM framework suggests that multiple cognitive processes contribute to task performance on a single trial, meaning that observed variations in RT and accuracy may reflect differences in multiple underlying processes rather than ability alone. If conventional interpretations were accurate, drift rate would consistently align with observed RT and accuracy, such that adverse experiences would have a similar impact on drift rate as conventional performance measures. However, if this relationship does not hold, it suggests that other cognitive processes, such as response caution or decision strategies, may be influenced more by adversity than general cognitive functioning. Further, given the prior findings of a general relationship between adversity and performance across diverse cognitive domains detailed above, drift rate's task‐general influence makes it a compelling mechanistic explanation for why adversity's impacts are so broadly reflected across cognitive tasks.

### 1.4 Current Study

The present study sought to address the gaps in literature by employing the DDM to characterize adolescent cognitive functioning in a perceptual emotional‐faces gender‐identification decision‐making task in a 15‐year longitudinal sample. This is the first longitudinal study to examine associations among childhood material hardship exposure, adolescent drift rate, and attentional problems. First, we examined whether cumulative exposure to material hardship across childhood (ages 1, 3, 5, and 9) was associated with drift rate in adolescence (age 15). Second, building upon the first aim, we tested whether the developmental trajectory of material hardship exposure across waves impacted drift rate. Third, we tested whether drift rate served as an underlying mechanism linking material hardship to attentional problems in adolescence. Finally, we assessed whether drift rate provided additional insights into cognitive processes beyond traditional behavioral measures, such as average RT and accuracy.

To distinguish the specific effects of household material hardship, our analyses adjusted for co‐occurring types of adversity—violence exposure and social deprivation—across childhood, in addition to demographic covariates. Additional sensitivity analyses examining the link between hardship and violence exposure are reported in the Supplement. We hypothesized that greater material hardship experience would be uniquely associated with lower adolescent drift rate (i.e., less efficient information processing). To address the aim of investigating material hardship changes across childhood, we used growth curve modeling to characterize latent trajectory of changes in hardship exposure across childhood and evaluated its link with drift rate. We hypothesized that an increase in material hardship over time would be associated with lower drift rate. With regards to links with attentional problems in adolescents, we hypothesized that lower drift rate would be associated with greater attentional problems in adolescents, and that drift rate would partially explain the association between material hardship exposure and attentional problems. Furthermore, we hypothesized that the associations we observe with drift rate would be distinct to the computational parameter and not observed when examining average RT and accuracy.

## 2 Methods

### 2.1 Sample and Procedure

Participants were recruited from the FFCWS, a population‐based longitudinal birth cohort study (Reichman et al. 2001). The original sample included 4898 children born in 20 large U.S. cities (population over 200,000), with an oversampling (3:1) for non‐marital births. This led to a sample of predominately unmarried and low‐income families. During adolescence (ages 15–17), a cohort of 237 families from midwestern cities (Detroit, MI; Toledo, OH; Chicago, IL) was invited to the University of Michigan to participate in the Study of Adolescent to Adult Neural Development (SAND) (Hein et al. 2018). Given the demographics of these cities, most youth in SAND identified as Black non‐Hispanic (76%), with 54% female. Adolescents and their primary caregivers completed a series of surveys and interviews, some of which are included in this study. Adolescents additionally completed behavioral tasks and magnetic resonance imaging (MRI) scans. Our analyses were restricted to those with usable performance data from the behavioral task, resulting in a final sample of 187 adolescents (see section on EEA below for exclusion details). Demographic characteristics are included in Table S1. The study was approved by the University of Michigan Institutional Review Board (IRB: HUM00167754; HUM0074392).

### 2.2 Measures

#### 2.2.1 Material Hardship

Longitudinal measures of household material hardship exposure during childhood were collected across four waves at ages 1, 3, 5, and 9. At each wave, caregivers indicated whether or not they had experienced hardship related to housing, utilities, food, healthcare, or finances within the last twelve months. Eight items that were repeatedly asked across waves were included in the analysis following a previous study (Hardi et al. 2022). These items were derived from the 1996 Survey of Income and Program Participation; the 1997 and 1999 New York City Social Indicators Survey; and the 1999 Study of Work, Welfare, and Family Wellbeing of Iowa families on Iowa's assistance program (Bauman 1999; Mayer and Jencks 1989).

At each wave, the primary caregiver responded yes (1) or no (0) on each of the following items: (1) received free food or meals, (2) evicted from home or apartment for not paying full rent/mortgage, (3) moved in with other people because of financial problems, (4) stay in a place not meant for long‐term housing (e.g., shelter, abandoned building, automobile), (5) did not receive medical care because of cost, (6) did not pay full gas/oil/electric bill, (7) did not pay full rent/mortgage, and (8) borrowed money from family/friends to pay bills. A sum score of material hardship experience was computed at each wave, with higher scores indicating greater material hardship. Cumulative household material hardship was computed as the sum of endorsed items across all waves. Correlations of hardship scores are reported in the Supplement.

#### 2.2.2 Adolescent Drift Rate (v)

Behavioral performance data for DDM parameter estimation was collected using a simple perceptual decision‐making task on emotional‐faces gender‐identification that was completed in an MRI scanner (Hardi et al. 2023; Hein et al. 2018, 2020). Participants were asked to identify the gender of the actor by pressing their thumb (male) or index finger (female) on a button box. Faces were selected from the NimStim set (Tottenham et al. 2009) and were counterbalanced for gender and race (White and Black). Each participant underwent 100 pseudo‐randomized trials with 5 types of emotional faces (20 faces per emotion): fearful, happy, angry, sad, and neutral. Each trial consisted of a 500 ms fixation cross, a 250 ms face stimuli, then a black screen of 1500 ms during which participants were instructed to respond to the face RTs and accuracy were recorded. Although the stimuli are emotionally‐salient, our analyses (see Supplement section on Emotion‐specific drift rate for supporting analyses) indicate that the specific parameters extracted here do not appear to be sensitive to the emotions being shown, and therefore we can still retain a theoretically valid estimate of drift rate from the performance data. Initial exploratory functional MRI analysis with contrast activation did not identify any reliable associations with drift rate (see Supplement for details).

A total of 201 youths completed the task. On average, participants completed almost all trials (98.8 trials), with an average accuracy of 93.8% and average RT of 644 ms. 13 participants were excluded due to poor performance: completed less than 75 trials (i.e., omission rate > 25%) and/or accuracy was below 55%, which might indicate that participants did not understand the task or were not engaged, resulting in 188 participants included in DDM modeling process. We additionally removed trials with RTs less than 200 ms as fast guesses, following standard procedures for fitting the DDM (Ratcliff and Tuerlinckx 2002). Because we were interested in the general cognitive abilities, we combined all trials for each participant without distinguishing between specific emotions. Doing so also preserved statistical power and stability of modeling given that the total trial numbers (max 100 trials per person) were below ideal number of trials for reliable estimations (200–400 trials) (Lerche et al. 2017; Voss et al. 2013).

**FIGURE 2:**
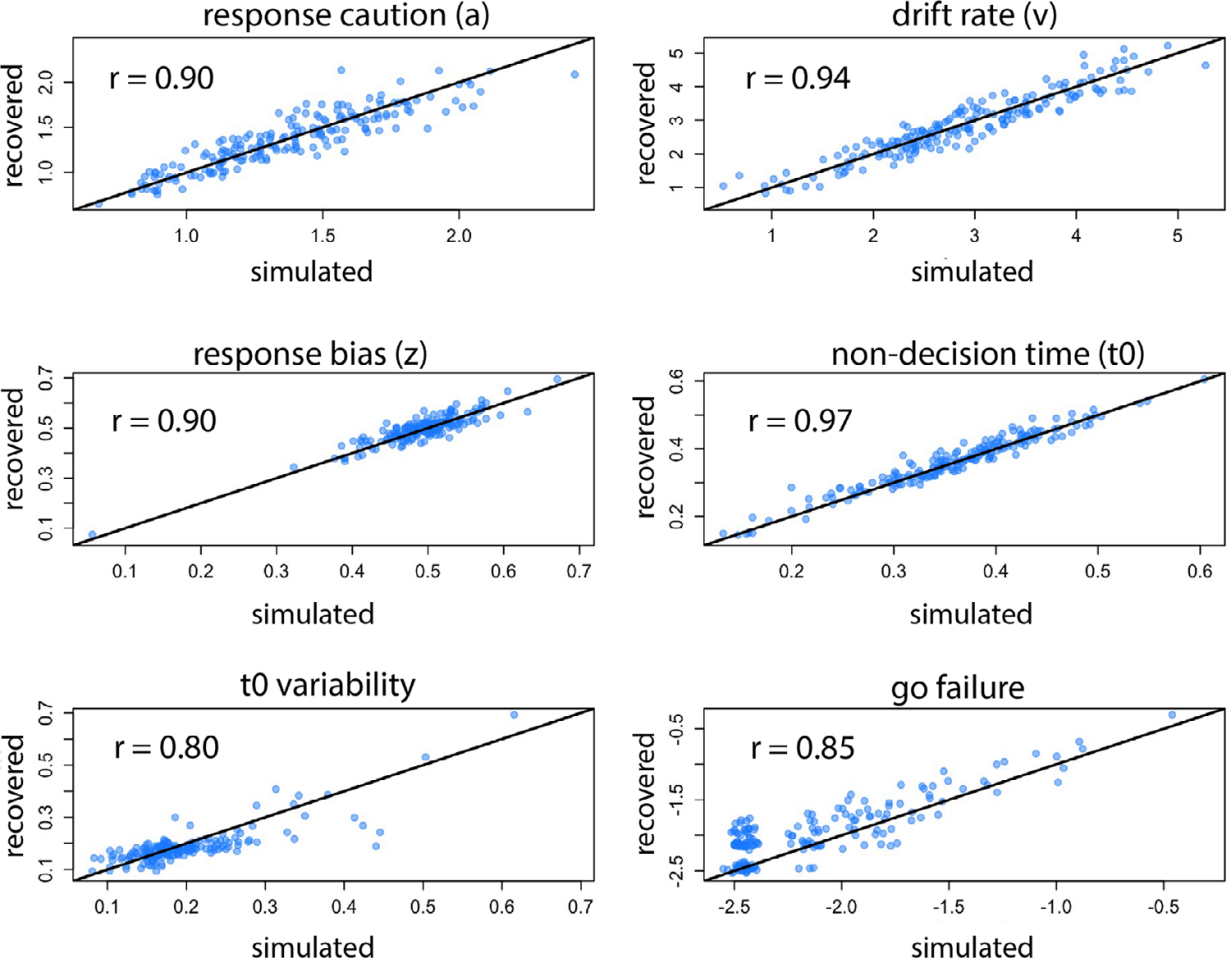
**Simulation‐recovery study correlation plots of DDM parameters**. Parameter values from the DDM (*x*‐axis) were used to simulate another data set, from which another set of parameter values were recovered (*y*‐axis). Correlation values (r) between the simulated and recovered parameter values are displayed on each plot. Diagonal lines indicate where the points would fall if the parameter recovery were perfect (r = 1). Parameter recovery for the four main parameters (top four panels: a, v, z, and t_0_) are excellent (rs >= 0.90), and the recovery for other estimated parameters are good (rs > 0.75).

DDM parameters were estimated using the Dynamic Models of Choice package in R (version 4.3.2, Heathcote et al. 2019; R Core Team 2023). Four main DDM parameters were estimated for each individual: drift rate (v), response caution (a), non‐decision time (t_0_), and response bias (z). One participant was further excluded based on poor model fit, resulting in a final sample of 187 participants. A simulation recovery study was conducted to show good recovery of DDM parameters (Figure 2). Point estimates of parameter values were summarized using the medians of posterior distributions (descriptives are shown in Table 1). Modeling details are reported in the Supplement.

**TABLE 1:** Distributions and zero‐order correlations of DDM parameters and raw performance data.

	*1*	*2*	*3*	*4*	*5*	*6*
*1. Drift rate (v)*	—	—	—	—	—	—
*2. Response Caution (a)*	−0.04	—	—	—	—	—
*3. Non‐decision Time (t0)*	**0.35** ^***^	**0.43** ^***^	—	—	—	—
*4. Response Bias (z)*	−0.01	0.06	0.01	—	—	—
*5. Reaction Time (second)*	−0.13	**0.81** ^***^	**0.66** ^***^	0.01	—	—
*6. Accuracy*	**0.49** ^***^	0.04	**0.31** ^***^	0.01	0.12	—
Mean	2.89	1.38	0.36	0.49	0.67	0.90
Standard deviation	0.91	0.33	0.08	0.05	0.13	0.19

^***^ *p* < 0.001.

#### 2.2.3 Adolescent Attentional Problems

We created multi‐informant, multi‐method measures of adolescent attentional problems using confirmatory factor analysis (CFA), which provides a more robust and less biased representations of the symptoms (De Los Reyes et al. 2015). Indicators in the CFA included 26 item‐level responses of parent‐reported from the Child Behavioral Checklist (CBCL) attention problems subscale (Achenbach and Rescorla 2001) and the clinician‐rated inattention and hyperactivity subscales from a DSM‐V semi‐structured clinical interview using the Kiddie Schedule for Affective Disorders and Schizophrenia (K‐SADS‐PL) (Kaufman et al. 1997). Details of the CFA are reported in the supplemental materials. The resulting latent factor in a community sample reflects dimensional variation in attention‐related difficulties, rather than a clinical diagnosis, with higher scores indicating greater symptoms, which in this sample reflects a dimension of typical individual differences in attention and impulsivity to more serious attention problems.

#### 2.2.4 Covariates

**Violence Exposure and Social Deprivation**: Composite scores of violence exposure and social deprivation were created based on procedures in previous studies (Goetschius et al. 2020; Hein et al. 2020). Briefly, violence exposure was assessed based on primary caregiver or mother report of direct child physical and emotional abuse, and indirect violence exposure (i.e., intimate partner and community violence) at child ages 3, 5, and 9. Social deprivation at child ages 3, 5, and 9 was created based on primary caregiver or mother report of physical and emotional neglect of the child, mother's intimate partner and community support. To compute the composite score, the zero‐centered scores of each item were summed and then divided by the number of items for each participant. The scores were then mean centered to create the final composite score, where greater score indicated more violence exposure and social deprivation.

**Demographic Covariates**: To account for potential confounding effects of material hardship on drift rate and drift rate on adolescent attentional problems, the following sociodemographic characteristics were controlled for in the analyses: sex, adolescent pubertal stage, race/ethnicity, and family structure. Sex was measured using parental report at child age 1 (0 = female, 1 = male). Adolescent pubertal stage was included as a covariate to account for potential differences in adolescent drift rate due to broad impacts of pubertal development in shaping neurocognitive features (Icenogle and Cauffman 2021). Pubertal stage was measured by youth report on the Pubertal Development Scale (Petersen et al. 1988), which asked about changes in child height, body hair, skin, facial hair, voice (male only), and breast development and menarche (female only). Responses were coded on a 4‐point scale (1 = no development, 4 = completed development) and the score was an average of all items endorsed. When adolescent report was not available, parent report was used. Ethnoracial identity was included as a covariate to adjust for potential effects of structural and interpersonal racism in shaping developmental experiences. Ethnoracial identity was self‐reported by adolescents (Black, non‐Hispanic; White, non‐Hispanic; Hispanic, and Other). The majority of participants in the sample identified as Black/African American (77.0%). Therefore, three dummy‐coded variables were created to represent ethnoracial identity with Black as the reference group. Finally, family structure was included as a covariate to adjust for potential differences in family functioning due to household composition. A household was identified as two‐parent (1) when the biological mother and father were cohabitating at baseline (child's birth), or were otherwise identified as single‐parent household (0).

#### 2.2.5 Statistical Analyses

**FIGURE 3:**
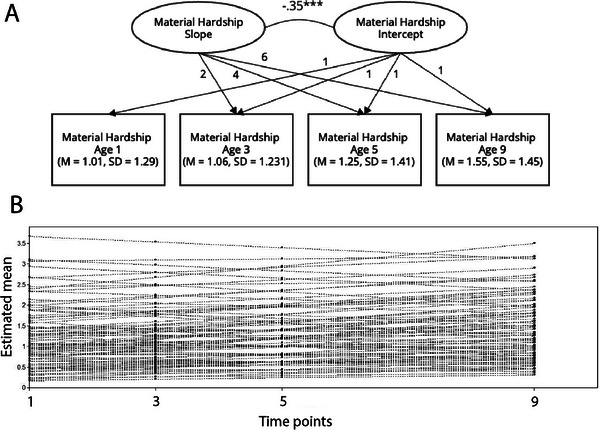
**Growth curve analysis with material hardship**. (A) Material hardship data at ages 1, 3, 5, and 9 from the Future of Families and Child Wellbeing study were used to improve estimations of growth curve model (*N* = 3714). Loadings of hardship estimating growth curve slope were fixed at 0, 2, 4, and 6. Estimated latent slopes and intercepts were significantly correlated and extracted for further analysis. (B) **Individual estimated growth curve trajectories**. Illustrating that material hardship increases with age. ***p < 0.001.

All statistical analyses were conducted in R Statistical Software v4.3.2 (R Core Team 2023) and MPlus v8.8 (Muthén and Muthén 2017). We accounted for missing data by using maximum likelihood estimations in regression analyses. For the first aim, we examined the bivariate correlation between cumulative material hardship and drift rate followed by a linear regression model adjusting for covariates. To investigate whether changes in the experiences of material hardship over time was associated with drift rate, we conducted a latent growth curve analysis in MPlus (details reported in Supplement). Estimated intercepts (initial time point) and slopes (change over time) for the subsample were then extracted to predict drift rate in a regression analysis in R (Figure 3).

For aim 3, we examined the bivariate correlation between drift rate and the latent factor of attentional problems, and regression with covariates was conducted. Further, we tested for the indirect effects of drift rate (i.e., drift rate as a mechanism linking material hardship exposure to attentional problems) using SPSS PROCESS macro package in R to calculate 95% confidence intervals with 10,000 bootstraps (Hayes 2021). Results were replicated in R Lavaan and MPlus. To examine the final aim, we investigated the same questions with behavioral performance metrics (i.e., average RT and accuracy) of the task instead of drift rate to compare the effects we observe from drift rate and conventional performance measures. Bivariate correlations between other parameters from DDM (i.e., a, t0, and z) with adversity items (i.e., material hardship, violence exposure, and social deprivation) and attentional problems are reported in the Supplement.

## 3 Results

### 3.1 Childhood Material Hardship Negatively Predicts Adolescent Drift Rate

**FIGURE 4:**
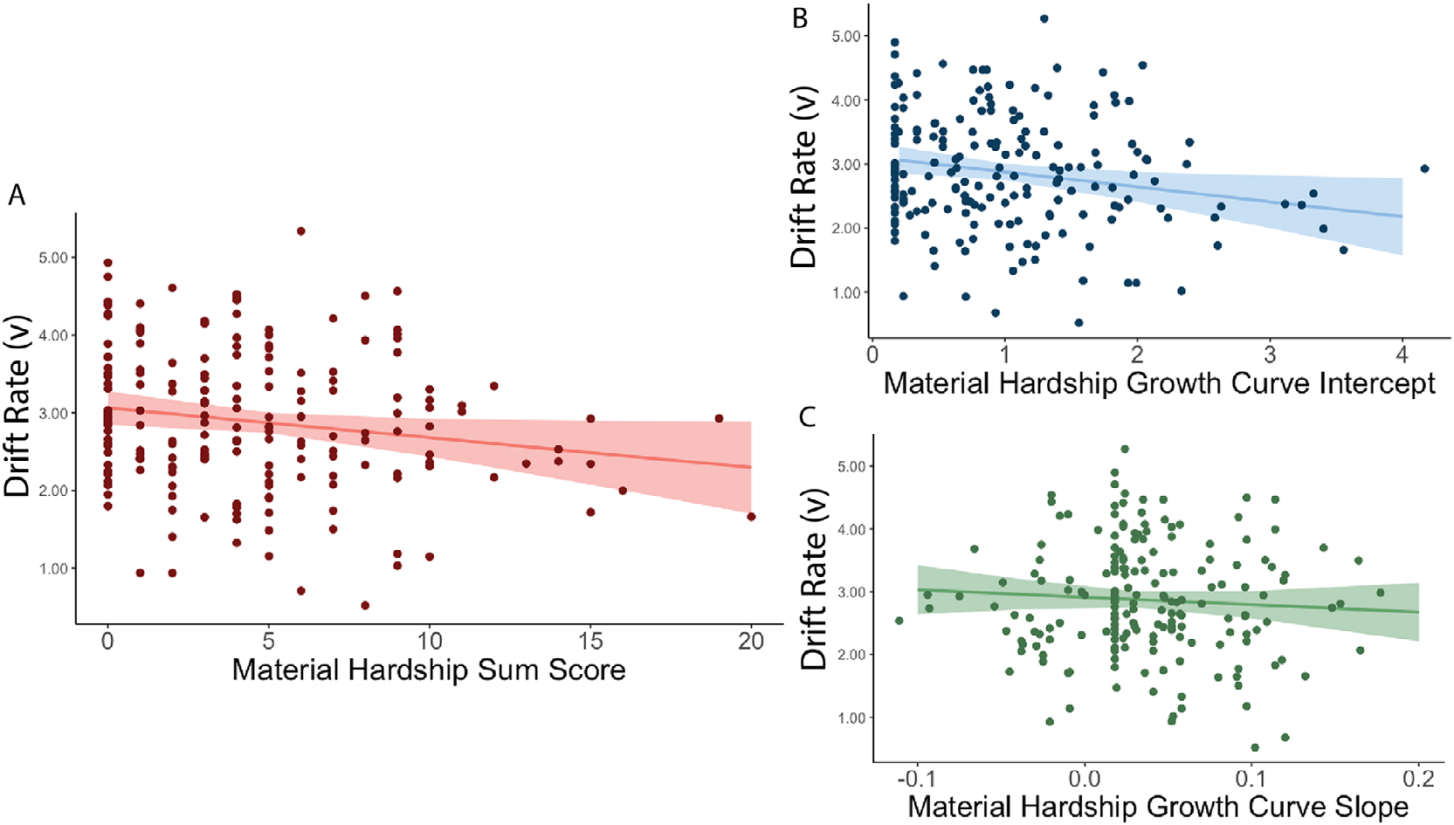
**Drift rate predicted by childhood material hardship**: (A) Cumulative material hardship negatively predicted adolescent drift rate. (B) Growth curve analysis with material hardship from ages 1, 3, 5, and 9 show that the intercept but not (C) the slope, was negatively associated with drift rate, suggesting that the initial exposure to material hardship at age 1, rather than changes in exposure across childhood, was more linked with adolescent drift rate.

Bivariate correlation between adolescent drift rate and cumulative material hardship showed a significant negative association (*r* = −0.17, *p* = 0.020). Regression analysis including covariates showed that the effect of cumulative material deprivation on drift rate survives after including other childhood adversity and demographic covariates in the model (*β* = −0.17, *p* = 0.038, Table 2, Figure 4). The results suggest that greater exposure to cumulative material hardship across childhood predicted less effective information processing during adolescent decision‐making (i.e., lower drift rate), above and beyond childhood experiences of violence exposure and social deprivation.

**TABLE 2:** Regression analysis of material hardship predicting drift rate (v).

*Predictors*	*b*	*β*	*SE*	*z*	p‐value
Model: v ∼ Material Hardship + Control Variables					
Material Hardship	−0.04	−0.17	0.02	−2.08	0.038^*^
Violence Exposure	−0.03	−0.02	0.15	−0.19	0.850
Social Deprivation	0.09	0.05	0.16	0.61	0.543
Sex	−0.16	−0.09	0.17	−0.97	0.331
Pubertal Age	0.12	0.08	0.15	0.86	0.389
Race: White	−0.07	−0.02	0.21	−0.33	0.742
Race: Hispanic	−0.10	−0.03	0.29	−0.35	0.730
Race: Other	0.28	0.07	0.30	0.98	0.325
Family Structure	−0.17	−0.09	0.14	−1.26	0.209

^*^ *p* < 0.05.

To further understand the effects of developmental trajectory of childhood material hardship on adolescent drift rate, we conducted a regression analysis using the intercept and slope extracted from growth curve analysis of material hardship to predict drift rate. Bivariate correlations show that the intercept of the growth curve (*r* = −0.17, *p* = 0.023) but not slope (*r* = −0.06, *p* = 0.534) was significantly associated with drift rate. Regression results when including intercept and slope in the same model show similar results (intercept: 𝛽 = −0.18, *p* = 0.013; slope 𝛽 = −0.08, *p* = 0.277). This suggests that the initial exposure to material hardship, rather than changes in exposure across time, had a stronger negative association with adolescent drift rate. Results remained significant in sensitivity analysis including other adversity and demographic covariates (intercept: *β* = −0.20, *p* = 0.017; slope: *β* = −0.07, *p* = 0.376) (Table 3, Figure 4).

**TABLE 3:** Regression analyses of material hardship growth curve slope and intercept predicting drift rate (v).

*Predictors*	*b*	*β*	*SE*	*z*	p‐value
Model 1: v ∼ i + s					
i (intercept)	−0.21	−0.18	0.09	−2.48	0.013^*^
s (slope)	−1.43	−0.08	1.31	−1.09	0.277
**Model 2: v ∼ i + s + Control Variables**					
i (intercept)	−0.23	−0.18	0.09	−2.36	0.017^*^
s (slope)	−1.19	−0.06	1.40	−0.77	0.376
Violence Exposure	−0.01	−0.01	0.15	−0.06	0.950
Social Deprivation	0.11	0.06	0.15	0.73	0.466
Sex	−0.15	−0.08	0.17	−0.87	0.382
Pubertal Age	0.12	0.08	0.14	0.85	0.397
Race: White	−0.07	−0.02	0.21	−0.33	0.744
Race: Hispanic	−0.13	−0.03	0.28	−0.46	0.644
Race: Other	0.30	0.07	0.29	1.04	0.300
Family Structure	−0.16	−0.09	0.13	−1.20	0.229

^*^ *p* < 0.05.

### 3.2 Adolescent Drift Rate Negatively Associated with Attentional Problems

As hypothesized, adolescent drift rate was negatively correlated with the latent factor of attentional problems (*r* = −0.18, *p* = 0.013), suggesting that less efficient information processing in decision‐making is associated with greater attentional problems in our sample of adolescents. Regression analysis (Table 4) revealed that the significant association between drift rate and attentional problems survived after controlling for demographic covariates (*β* = −0.15, *p* = 0.040).

**TABLE 4:** Regression analyses of drift rate (v) and attentional problems latent factor.

*Predictors*	*b*	*β*	*SE*	*z*	p‐value
Model: Attentional problems ∼ v + Demographic control variables					
v	−0.13	−0.15	0.06	−2.04	0.041^*^
Sex	0.25	0.16	0.14	1.76	0.079
Pubertal Age	−0.14	−0.11	0.12	−1.18	0.238
Race: White	0.03	0.01	0.17	0.15	0.883
Race: Hispanic	0.15	0.04	0.25	0.58	0.560
Race: Other	0.27	0.08	0.24	1.09	0.275
Family Structure	−0.08	−0.05	0.12	−0.73	0.468

^*^ *p* < 0.05.

**FIGURE 5:**
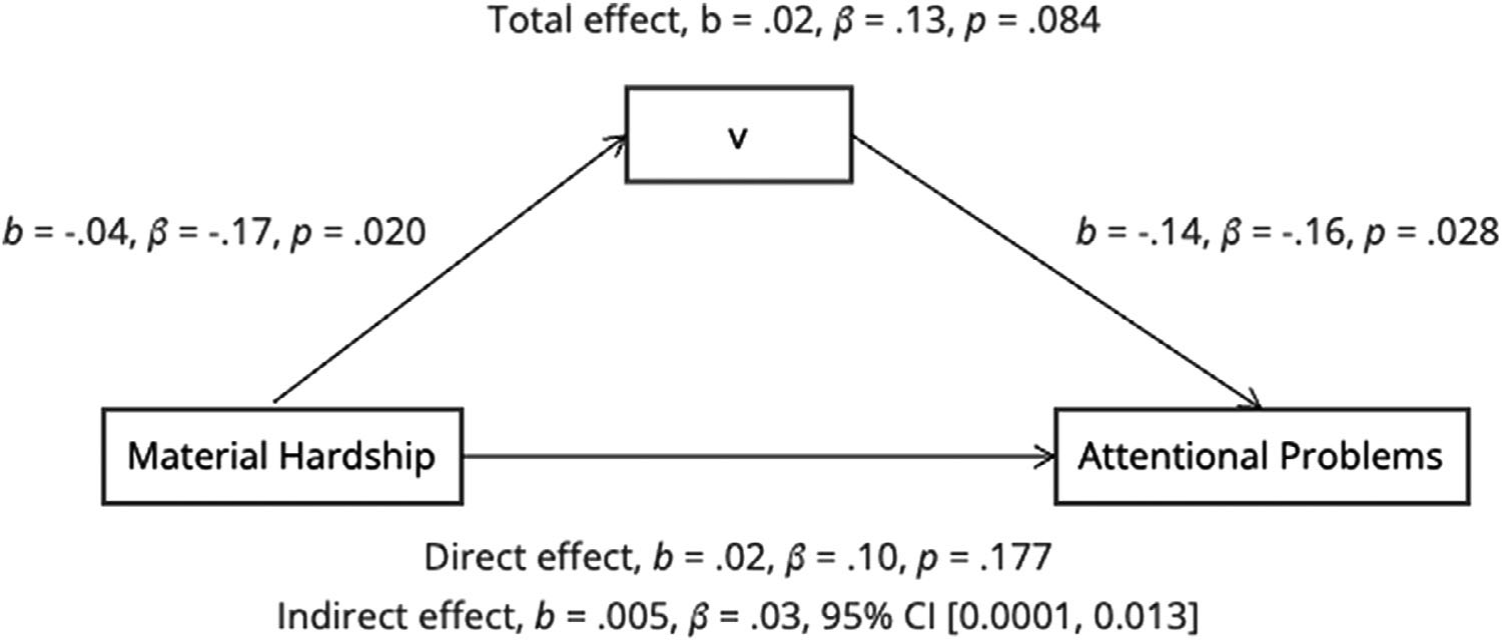
**Indirect effects model of material hardship on attentional problems via drift rate (v)**. **T**he total effect of material hardship and drift rate on attentional problems was not significant (*b* = 0.02, *t* = 1.74, *p* = 0.084). We found a small but significant indirect effect of hardship exposure on adolescent attentional problems via drift rate (𝛽 = 0.03, b = 0.005, 95% CI [0.0001, 0.013]).

The model of indirect effects of drift rate linking material hardship exposure to attentional problems is shown in Figure 5. The direct effects model was not significant (*F* (1,184) = 3.03, *p* = 0.084). However, we did observe a small indirect effect of hardship exposure on adolescent attentional problems via drift rate (𝛽 = 0.03, b = 0.005, 95% CI [0.0001, 0.013]). Individuals who experienced greater childhood material hardship showed lower drift rate in adolescence, which in turn, was associated with greater attentional problems.

### 3.3 Comparisons with Conventional Performance Metrics

To better understand the results we observed from analyses with drift rate, we also conducted the analyses with raw performance metrics: average RTs and average accuracy. First, we found that the correlations between drift rate and behavioral measures were in the expected directions (Table 1): greater drift rate (i.e., more efficient decision‐making) was associated with greater accuracy (*r* = 0.49, *p* < 0.001) and faster RT (though the correlation was not significant: *r* = −0.13, *p* = 0.080). At the same time, we did not find the same significant associations between material hardship with average RT (*r* = 0.04, *p* = 0.551) or average accuracy (*r* = −0.12, *p* = 0.095). We also did not replicate the association between raw performance metrics and attentional problems (average RT: *r* = −0.04, *p* = 0.540; average accuracy: *r* = −0.03, *p* = 0.792). The null results with conventionally used behavioral metrics suggest that drift rate can help reveal specific cognitive processes and the relations with adversity and behavioral problems that would otherwise not be detected.

## 4 Discussion

This study examined the association between childhood material hardship and adolescent evidence accumulation efficiency (i.e., drift rate, v), a computational underpinning of higher‐order cognitive functioning that is hypothesized to be explained by a task‐general factor of cognitive efficiency EEA. Our results showed that greater cumulative material hardship across childhood was associated with lower adolescent drift rate, above and beyond exposure to violence, social deprivation, and other demographic covariates. We also found that lower adolescent drift rate was associated more attentional problems. In addition, we observed a small significant indirect effect between hardship and attention problems via drift rate. To our knowledge, this is the first study to leverage longitudinal data to investigate cognitive consequences of material hardship, a measure of the lived experiences of poverty that may be a more accurate assessment of socioeconomic disadvantage. Additionally, the application of DDM to examine adversity‐related differences in cognitive processing contributes to the growing body of research utilizing computational methods to address developmental questions. By moving beyond conventional task performance metrics, the DDM decomposes trial‐wise behavior into distinct and meaningful cognitive processes, offering a more nuanced understanding of how childhood material hardship is associated with cognitive functioning in adolescents.

### 4.1 Childhood Material Hardship and Adolescent Cognitive Efficiency

Our results showed that exposure to material hardship, but not violence exposure or social deprivation, was associated with drift rate. This finding largely aligns with past research showing that economic deprivation specifically is associated with lower cognitive performance (Schenck‐Fontaine and Ryan 2022; Young et al. 2022). However, our findings contrasted with prior research specifically examining drift rate or EEA as a cognitive outcome, which reported that greater violence exposure, rather than material hardship, was associated with lower general EEA across several cognitive tasks (Vermeent, Young, Gelder, et al. 2024). The inconsistencies in results could be due to a variety of reasons, such as the use of cross‐sectional designs rather than longitudinal approach used in our study, differences in samples, including demographic or socioeconomic characteristics, variations in the severity or timing of exposure to adversities, or differences in the selection of tasks used to estimate EEA. In addition, the material hardship measure used in Vermeent, Young, Gelder, et al. (2024) was a factor score that adjusted for demographic variables, which contrasts with our use of sum scores of hardship endorsements and could have contributed to the difference in results. Given that this question is only beginning to be researched, more well‐sampled studies with computational models of cognition and longitudinal designs are needed to replicate these results and clarify the nuanced effects of distinct types of adversity on cognitive processes like EEA.

Using growth curve analysis, we also investigated how initial exposure to material hardship (i.e., intercept) and changes in trajectory of experience across childhood (i.e., slope) linked with drift rate. We found that greater initial exposure to hardship at age 1, but not trajectory of change in hardship levels, was associated with lower drift rate. This finding suggests that baseline level of material hardship experience resulted in long‐lasting impact on cognitive performance, whereas the increase in hardship over time may be less influential compared with initial conditions. This finding is somewhat inconsistent with past research showing that decrease in parental income over time is associated with poorer cognitive outcomes, as summarized by a recent review (Levesque et al. 2021). However, there is evidence supporting the idea that early economic disadvantage can have enduring negative effects on mental health outcomes in children, potentially due to persistent proximal experiences and social interactions that remain unchanged regardless of later economic changes (Caspi et al. 1989; McLeod and Shanahan 1996). A similar explanation may apply to our findings: early household material hardship could have contributed to long lasting stressful conditions in the surrounding environment, such as tension in family relationships. Such chronic stress exposure has been linked with impairments in executive functioning regions in the brain (e.g., Liston et al. 2009), which could contribute to poorer cognitive performance in adolescence (Hackman et al. 2010). In addition, material hardship experiences may also involve a lack of nutrition and quality health care, which could impact the neural mechanisms underlying cognition (e.g., Gómez‐Pinilla 2008). While our study does not directly test what these mechanisms might be, future research should aim to clarify these pathways by examining the interplay between early adversity, stress levels, and cognitive development over time.

Our finding may be explained by theories emphasizing the role of cognitive stimulation in the environment as a critical factor in promoting cognitive development (Hackman et al. 2015; Rakesh et al. 2024; Rosen et al. 2020). Cognitively stimulating environments—those that offer a variety of stimuli (e.g., books and toys) that encourage knowledge‐building—support exploration and learning, which are essential for cognitive development across domains (Vygotsky 1978). In households experiencing material hardship and struggling to meet basic needs, providing such environments may be particularly challenging. Evidence from both human intervention studies and animal models suggests that enriched environments not only enhance cognitive outcomes but also support underlying neural functioning (Jankowsky et al. 2005; Mandolesi et al. 2017; McLoyd 1998). In households experiencing material hardship and struggling to meet basic needs, providing such cognitive‐stimulating environments may be particularly challenging, potentially limiting opportunities for cognitive development.

Alternatively, our results can also be interpreted through an adaptive lens, such that lower drift rate may reflect developmentally adjusted cognitive styles that are potentially advantageous in unpredictable or resource‐scarce environments. Consistent with the hidden talents framework, adversity‐exposed individuals may exhibit cognitive adaptations that are context‐ and content‐dependent, thereby showing strengths in domains that are ecologically relevant despite differences on conventional cognitive tasks (Ellis et al. 2022; Mittal et al. 2015; Young et al. 2022). Recent theoretical accounts also proposed that individuals exposed to adversity may engage in evidence accumulation less selectively across competing information sources (Niebaum et al. 2025). While we did not find any evidence for adversity exposure impacting other facets of decision‐making (i.e., response caution, non‐decision time, and bias), previous work has found that adversity may impact response caution as well (Vermeent et al. 2024). Future work should examine whether these cognitive profiles confer functional advantages in real‐world settings and explore how different environmental demands shape the developmental trajectory of decision‐making processes.

### 4.2 Drift Rate and Adolescent Attentional Problems

We found evidence that lower drift rate is linked with greater attentional problems in adolescents. This finding aligns with past clinical research showing individuals with ADHD consistently displayed lower EEA (Huang‐Pollock et al. 2017; Weigard and Huang‐Pollock 2017; Ziegler et al. 2016). Our finding also aligns with the growing body of research highlighting reductions in EEA across various domains, including perceptual decision‐making (as measured by our task), attention, and inhibition, among others. We also observed a statistically significant indirect effect of childhood material hardship on adolescent attentional problems via drift rate. Although the total and direct effects of material hardship on attentional problems were not statistically significant, this finding suggests that early life material hardship may influence reduced cognitive efficiency, which in turn may influence attentional functioning in adolescence. However, the effect size of the indirect effect is small, suggesting that though significant, drift rate only explains a small proportion of the effect. At the same time, given the partially cross‐sectional nature of our data and the lack of direct and total effects (i.e., drift rate and attention were measured concurrently), we interpret this indirect pathway cautiously and do not make claims about temporal or causal mediation. Future longitudinal studies with repeated assessments of both cognition and behavior will be critical for clarifying the directionality and developmental timing of these associations.

### 4.3 Drift Rate Reflects Specific Cognitive Process

We conducted the same analyses as above with conventional behavior performance metrics: average RT and accuracy. Although drift rate was significantly correlated with both measures in the expected directions, we did not see the same findings with either material hardship or attentional problems with conventional performance measures. This is consistent with simulation studies (Stafford et al. 2020) and past research showing that drift rate differentiates behavioral responses in individuals with high versus low anxiety, whereas accuracy and RT did not capture the same effects (White et al. 2010). Similar findings have also been observed in neural patterns, where Ho and colleagues (2014) found significant activation in face‐processing neural regions associated with processing efficiency (estimated similarly to EEA) in depressed individuals, but did not observe this effect with mean RT or accuracy. We also did not observe any significant associations with hardship exposure, or other types of adversity experiences, with other parameters from the DDM model, highlighting the specificity of drift rate in understanding the impact of socioeconomic disadvantage. In addition, we found that greater attentional difficulties in adolescents were linked with slower non‐decision time, which aligns with past research and may suggest that individuals with attentional problems may exhibit faster encoding and/or motor responses that are not associated with the decision‐process, although the specific processes are not separable in the context of DDM (Karalunas et al. 2014). Taken together, these results underscore the value of computational models in assessing cognitive performance. These findings also suggest that although task performance is related to cognitive ability (as measured by drift rate or EEA), important differences exist that could lead to problematic inferences when using these conventional measures as proxies for cognition. Therefore, future studies should consider incorporating computational models to provide a more accurate and reliable assessment of cognitive performance.

### 4.4 Strengths, Limitations, and Future Directions

The current study has multiple strengths. First, the data is drawn from a 15‐year longitudinal dataset in which adversity measures were collected prospectively at each assessment point. Second, the use of a computational model of cognition allows for a more nuanced understanding of cognitive processes, offering a novel and theoretically meaningful approach compared to conventional behavioral measures in addressing developmental questions. Third, our dataset allowed for creation of a multi‐informant measure of attentional problems, which provides a more comprehensive and accurate account of symptoms based on reports from both parents and clinicians.

The present study also has several limitations. First, our sample size was modest for examining longitudinal associations between material hardship, drift rate, and attentional problems in adolescents (*N* = 187). While we did not conduct an a‐priori power analysis, future research should aim to replicate these findings with larger samples given the small effects observed. Second, our study only included a single two‐choice perceptual decision‐making task that allowed for application of computational modeling. Although this limits our ability to construct a latent task‐general EEA factor, prior research has shown that drift rate estimates derived from individual tasks are reasonable approximations of the general underlying cognitive process due to their strong psychometric properties (Löffler et al. 2024; Schubert et al. 2016; Tomlinson et al. 2025; Weigard et al. 2021). Thus, although we can interpret our single‐task drift rate estimate as representing EEA, we acknowledge that it likely contains some task‐specific variance. Notably, past studies have also shown that task‐specific and task‐general EEA may differ in their susceptibility to adversity‐related influences (Vermeent et al. 2024), highlighting the value of examining both in future work. In addition, the current task involves emotionally‐salient stimuli that may engage affective processing systems differently in individuals and interact with the decision processes in various ways. Despite our efforts to account for valence by modeling emotion‐specific drift rate, these subtle patterns may not be fully captured by the current modeling approaches. The task was also conducted in a lab‐based, MRI scanner environment, which could have an impact on the participants’ cognitive performance. Given these task‐level limitations, our study results should be interpreted with caution. To provide a more comprehensive understanding, future studies should include a range of tasks that measure different cognitive processes (e.g., perceptual processing, attention, inhibition), as well as across different contexts (e.g., valence) to construct more robust latent measures of task‐general and task‐specific EEA. Third, our primary measure of material hardship was reported by the primary caregiver for the entire household. As a result, we were unable to distinguish between the parent's exposure to hardship and its direct impact on the child. For example, it is possible that while parents may not have enough food for themselves, they still manage to provide meals for their children, meaning the child may not directly experience food insecurity. Although research suggests that both direct and indirect experiences of material hardship can lead to negative developmental outcomes (Conger et al. 2010; McCarthy et al. 2018), future studies should explore whether differences between these forms of hardship exist and how they differentially affect child development.

Results from the present study open several avenues for further exploration. Our study provides initial support that cumulative material hardship is associated with drift rate, but the mechanism of this association remains unclear. Studies could explore this from several directions, including investigating the level of cognitive stimulation in the environment. In addition, studies could also explore the biological underpinnings of this associations. One promising link could be alterations in white matter, which has been linked to both material hardship (Hardi et al. 2022) and cognitive functioning (Ferrer et al. 2013). More personalized methods in capturing functional connectivity patterns of EEA could also better elucidate the underlying neural mechanisms compared with activation‐based analyses that generalizes across neural activity. Such analyses, ideally with larger, well‐powered samples, could help elucidate how adversity impacts cognitive efficiency through structural and functional brain functioning. Furthermore, while our study replicated previous findings that drift rate is associated with attention difficulties, EEA has also been proposed as a transdiagnostic neurocognitive risk factor for various forms of psychopathology (Weigard and Sripada 2021). Future research should test this hypothesis by examining differences in EEA between clinical and non‐clinical populations to better understand its potential as a broad marker of risk across mental health conditions.

### 4.5 Conclusion

Taken together, our study applied diffusion‐decision modeling to investigate longitudinal associations between material hardship and cognition in a sample of adolescents traditionally underrepresented in research. We found that material hardship, beyond other forms of adversity such as exposure to threat and social deprivation, significantly influences adolescent drift rate, which is further associated with attentional problems. These findings support drift rate (and EEA) as a mechanistic explanation for the broad effects of material hardship across cognitive domains and underscore the unique advantages of using computational models of cognition, compared to conventional performance measures, in uncovering associations with environmental risk factors. By identifying the specific ways in which adversity influences cognitive development, such research could guide interventions and policies aimed at mitigating these effects, such as policies that bolster family resources or enhance access to cognitively enriching opportunities.

## Author Contributions

**Yue Linda Zhang**: conceptualization, data curation, formal analysis, methodology, project administration, software, visualization, writing – original draft, writing – review and editing, validation. **Alexander S. Weigard**: conceptualization, funding acquisition, methodology, software, writing – review and editing. **Felicia A. Hardi**: conceptualization, writing – review and editing, formal analysis. **Sunghyun H. Hong**: conceptualization, writing – review and editing. **Edward Huntley**: resources, writing – review and editing, conceptualization. **Colter Mitchell**: conceptualization, funding acquisition, writing – review and editing, project administration, resources. **Luke W. Hyde**: conceptualization, funding acquisition, resources, writing – review and editing, supervision. **Christopher S. Monk**: supervision, conceptualization, funding acquisition, writing – review and editing, project administration, resources.

## Funding

The authors have nothing to report.

## Conflicts of Interest

The authors declare no conflicts of interest.

## Ethics Statement

This study was approved by the University of Michigan Institutional Review Board. The procedures used in this study adhere to the tenets of the Declaration of Helsinki.

## Supporting information



Supporting Material: desc70213‐sup‐0001‐SuppMat.docx

## Data Availability

Data will be openly available at https://nda.nih.gov/edit_collection.html?id=2106.

## References

[desc70213-bib-0001] Achenbach, T. M. , and L. A.l Rescorla . 2001. Manual for the ASEBA School‐Age Forms & Profiles. University of Vermont, Research Center for Children, Youth & Families.

[desc70213-bib-0002] Alaimo, K. , C. M. Olson , and E. A. Frongillo Jr . 2001. “Food Insufficiency and American School‐Aged Children's Cognitive, Academic, and Psychosocial Development.” Pediatrics 108, no. 1: 44–53. 10.1542/peds.108.1.44. 11433053

[desc70213-bib-0003] Bauman, K. J. 1999. “Shifting Family Definitions: The Effect of Cohabitation and Other Nonfamily Household Relationships on Measures of Poverty.” Demography 36, no. 3: 315–325. 10.2307/2648055. 10472496

[desc70213-bib-0004] Caspi, A. , D. J. Bem , and G. H. Elder Jr . 1989. “Continuities and Consequences of Interactional Styles across the Life Course.” Journal of Personality 57, no. 2: 375–406. 10.1111/j.1467-6494.1989.tb00487.x. 2769561

[desc70213-bib-0005] Conger, R. D. , K. J. Conger , and M. J. Martin . 2010. “Socioeconomic Status, Family Processes,and Individual Development.” Journal of Marriage and Family 72, no. 3: 685–704. 10.1111/j.1741-3737.2010.00725.x. 20676350 PMC2910915

[desc70213-bib-0006] R Core Team . 2023. “R: A Language and Environment for Statistical Computing.” R Foundation for Statistical Computing. https://www.R‐project.org/.

[desc70213-bib-0007] De Los Reyes, A. , T. M. Augenstein , M. Wang , et al. 2015. “The Validity of the Multi‐Informant Approach to Assessing Child and Adolescent Mental Health.” Psychological Bulletin 141, no. 4: 858–900. 10.1037/a0038498. 25915035 PMC4486608

[desc70213-bib-0008] de Zeeuw, E. L. , J.‐J. Hottenga , K. G. Ouwens , et al. 2020. “Intergenerational Transmission of Education and ADHD: Effects of Parental Genotypes.” Behavior Genetics 50, no. 4: 221–232. 10.1007/s10519-020-09992-w. 32026073 PMC7355279

[desc70213-bib-0097] Dellefratte, K. , J. A. Stingone , and L. Claudio . 2019. “Combined Association of BTEX and Material Hardship on ADHD‐Suggestive Behaviours Among a Nationally Representative Sample of US Children.” Paediatric and Perinatal Epidemiology 33, no. 6: 482–489. 10.1111/ppe.12594. 31657027 PMC7092642

[desc70213-bib-0009] Diamond, A. 2013. “Executive Functions.” Annual Review of Psychology 64, no. 1: 135–168. 10.1146/annurev-psych-113011-143750. PMC4084861 23020641

[desc70213-bib-0010] Duyser, F. A. , P. F. V. Eijndhoven , R. M. Collard , et al. 2025. “Measuring Self‐Referent Memory Bias as Marker for Depression: Overview, New Insights, and Recommendations.” Journal of Psychopathology and Behavioral Assessment 47, no. 1: 10. 10.1007/s10862-024-10190-9.

[desc70213-bib-0011] Eisenberg, I. W. , P. G. Bissett , A. Zeynep Enkavi , et al. 2019. “Uncovering the Structure of Self‐regulation Through Data‐Driven Ontology Discovery.” Nature Communications 10, no. 1: 2319. 10.1038/s41467-019-10301-1. PMC6534563 31127115

[desc70213-bib-0012] Ellis, B. J. , L. S. Abrams , A. S. Masten , R. J. Sternberg , N. Tottenham , and W. E. Frankenhuis . 2022. “Hidden Talents in Harsh Environments.” Development and Psychopathology 34, no. 1: 95–113. 10.1017/S0954579420000887. 32672144

[desc70213-bib-0013] Evans, G. W. , D. Li , and S. S. Whipple . 2013. “Cumulative Risk and Child Development.” Psychological Bulletin 139, no. 6: 1342–1396. 10.1037/a0031808. 23566018

[desc70213-bib-0014] Farah, M. J. , D. M. Shera , J. H. Savage , et al. 2006. “Childhood Poverty: Specific Associations With Neurocognitive Development.” Brain Research 1110, no. 1: 166–174. 10.1016/j.brainres.2006.06.072. 16879809

[desc70213-bib-0015] Feinstein, L. , and J. Bynner . 2004. “The Importance of Cognitive Development in Middle Childhood for Adulthood Socioeconomic Status, Mental Health, and Problem Behavior.” Child Development 75, no. 5: 1329–1339. 10.1111/j.1467-8624.2004.00743.x. 15369517

[desc70213-bib-0016] Ferrer, E. , K. J. Whitaker , J. S. Steele , C. T. Green , C. Wendelken , and S. A. Bunge . 2013. “White Matter Maturation Supports the Development of Reasoning Ability through Its Influence on Processing Speed.” Developmental Science 16, no. 6: 941–951. 10.1111/desc.12088. 24118718 PMC3801426

[desc70213-bib-0017] Fields, A. , P. A. Bloom , M. VanTieghem , et al. 2021. “Adaptation in the Face of Adversity: Decrements and Enhancements in Children's Cognitive Control Behavior Following Early Caregiving Instability.” Developmental Science 24, no. 6: e13133. 10.1111/desc.13133. 34080760 PMC8530827

[desc70213-bib-0018] Gershoff, E. T. , J. L. Aber , C. C. Raver , and M. C. Lennon . 2007. “Income Is Not Enough: Incorporating Material Hardship Into Models of Income Associations with Parenting and Child Development.” Child Development 78, no. 1: 70–95. 10.1111/j.1467-8624.2007.00986.x. 17328694 PMC2835994

[desc70213-bib-0019] Goetschius, L. G. , T. C. Hein , C. Mitchell , et al. 2020. “Childhood Violence Exposure and Social Deprivation Predict Adolescent Amygdala‐Orbitofrontal Cortex White Matter Connectivity.” Developmental Cognitive Neuroscience 45: 100849. 10.1016/j.dcn.2020.100849. 32890959 PMC7481532

[desc70213-bib-0020] Gómez‐Pinilla, F. 2008. “Brain Foods: The Effects of Nutrients on Brain Function.” Nature Reviews. Neuroscience 9, no. 7: 568–578. 10.1038/nrn2421. PMC2805706 18568016

[desc70213-bib-0021] Hackman, D. A. , M. J. Farah , and M. J. Meaney . 2010. “Socioeconomic Status and the Brain: Mechanistic Insights From Human and Animal Research.” Nature Reviews Neuroscience 11, no. 9: 651–659. 10.1038/nrn2897. 20725096 PMC2950073

[desc70213-bib-0022] Hackman, D. A. , R. Gallop , G. W. Evans , and M. J. Farah . 2015. “Socioeconomic Status and Executive Function: Developmental Trajectories and Mediation.” Developmental Science 18, no. 5: 686–702. 10.1111/desc.12246. 25659838

[desc70213-bib-0023] Hanson, J. L. 2025. “Stress About Eviction or Loss of Housing and Child Mental Health.” JAMA Network Open 8, no. 2: e2458984. 10.1001/jamanetworkopen.2024.58984. 39937477 PMC11822551

[desc70213-bib-0024] Hardi, F. A. , L. G. Goetschius , V. McLoyd , et al. 2023. “Adolescent Functional Network Connectivity Prospectively Predicts Adult Anxiety Symptoms Related to Perceived COVID‐19 Economic Adversity.” Journal of Child Psychology and Psychiatry 64, no. 6: 918–929. 10.1111/jcpp.13749. 36579796 PMC9880614

[desc70213-bib-0025] Hardi, F. A. , L. G. Goetschius , M. K. Peckins , et al. 2022. “Differential Developmental Associations of Material Hardship Exposure and Adolescent Amygdala–Prefrontal Cortex White Matter Connectivity.” Journal of Cognitive Neuroscience 34, no. 10: 1866–1891. 10.1162/jocn_a_01801. 34942644 PMC9651170

[desc70213-bib-0026] Hardi, F. A. , M. K. Peckins , C. Mitchell , et al. 2024. “Childhood Adversity and Adolescent Mental Health: Examining Cumulative and Specificity Effects Across Contexts and Developmental Timing.” Development and Psychopathology 1–17. 10.1017/S0954579424001512. 39359017 PMC11965435

[desc70213-bib-0027] Haushofer, J. , and E. Fehr . 2014. “On the Psychology of Poverty.” Science 344, no. 6186: 862–867. 10.1126/science.1232491. 24855262

[desc70213-bib-0028] Hayes, A. F. 2021. Introduction to Mediation, Moderation, and Conditional Process Analysis: Third Edition: a Regression‐Based Approach. Guilford Press. https://www.guilford.com/books/Introduction‐to‐Mediation‐Moderation‐and‐Conditional‐Process‐Analysis/Andrew‐Hayes/9781462549030.

[desc70213-bib-0029] He, X. , B. Qiu , Y. Deng , et al. 2024. “Material Hardship Predicts Response Bias in Loss‐Averse Decisions: The Roles of Anxiety and Cognitive Control.” The Journal of Psychology 158, no. 4: 309–324. 10.1080/00223980.2023.2296946. 38227200

[desc70213-bib-0030] Heathcote, A. , Y.‐S. Lin , A. Reynolds , L. Strickland , M. Gretton , and D. Matzke . 2019. “Dynamic Models of Choice.” Behavior Research Methods 51, no. 2: 961–985. 10.3758/s13428-018-1067-y. 29959755

[desc70213-bib-0031] Hedge, C. , G. Powell , and P. Sumner . 2018. “The Reliability Paradox: Why Robust Cognitive Tasks Do Not Produce Reliable Individual Differences.” Behavior Research Methods 50, no. 3: 1166–1186. 10.3758/s13428-017-0935-1. 28726177 PMC5990556

[desc70213-bib-0032] Hein, T. C. , L. G. Goetschius , V. C. McLoyd , et al. 2020. “Childhood Violence Exposure and Social Deprivation Are Linked to Adolescent Threat and Reward Neural Function.” Social Cognitive and Affective Neuroscience 15, no. 11: 1252–1259. 10.1093/scan/nsaa144. 33104799 PMC7745142

[desc70213-bib-0033] Hein, T. C. , W. I. Mattson , H. L. Dotterer , et al. 2018. “Amygdala Habituation and Uncinate Fasciculus Connectivity in Adolescence: A Multi‐Modal Approach.” Neuroimage 183: 617–626. 10.1016/j.neuroimage.2018.08.058. 30172004 PMC6197897

[desc70213-bib-0035] Huang, Y. , C. M. Heflin , and A. Validova . 2021. “Material Hardship, Perceived Stress, and Health in Early Adulthood.” Annals of Epidemiology 53: 69–75.e3. 10.1016/j.annepidem.2020.08.017. 32949721 PMC7494502

[desc70213-bib-0036] Huang‐Pollock, C. , R. Ratcliff , G. McKoon , Z. Shapiro , A. Weigard , and H. Galloway‐Long . 2017. “Using the Diffusion Model to Explain Cognitive Deficits in Attention Deficit Hyperactivity Disorder.” Journal of Abnormal Child Psychology 45, no. 1: 57–68. 10.1007/s10802-016-0151-y. 27030470 PMC5045756

[desc70213-bib-0037] Icenogle, G. , and E. Cauffman . 2021. “Adolescent Decision Making: A Decade in Review.” Journal of Research on Adolescence 31, no. 4: 1006–1022. 10.1111/jora.12608. 34820945

[desc70213-bib-0038] Jankowsky, J. L. , T. Melnikova , D. J. Fadale , et al. 2005. “Environmental Enrichment Mitigates Cognitive Deficits in a Mouse Model of Alzheimer's Disease.” Journal of Neuroscience 25, no. 21: 5217–5224. 10.1523/JNEUROSCI.5080-04.2005. 15917461 PMC4440804

[desc70213-bib-0039] Johnson, D. , J. Policelli , M. Li , et al. 2021. “Associations of Early‐Life Threat and Deprivation With Executive Functioning in Childhood and Adolescence: A Systematic Review and Meta‐Analysis.” JAMA Pediatrics 175, no. 11: e212511. 10.1001/jamapediatrics.2021.2511. 34309651 PMC8314173

[desc70213-bib-0040] Jyoti, D. F. , E. A. Frongillo , and S. J. Jones . 2005. “Food Insecurity Affects School Children's Academic Performance, Weight Gain, and Social Skills.” Journal of Nutrition 135, no. 12: 2831–2839. 10.1093/jn/135.12.2831. 16317128

[desc70213-bib-0041] Karalunas, S. L. , H. M. Geurts , K. Konrad , S. Bender , and J. T. Nigg . 2014. “Annual Research Review: Reaction Time Variability in ADHD and Autism Spectrum Disorders: Measurement and Mechanisms of a Proposed Trans‐Diagnostic Phenotype.” Journal of Child Psychology and Psychiatry 55, no. 6: 685–710. 10.1111/jcpp.12217. 24628425 PMC4267725

[desc70213-bib-0042] Kaufman, J. , B. Birmaher , D. Brent , et al. 1997. “Schedule for Affective Disorders and Schizophrenia for School‐Age Children‐Present and Lifetime Version (K‐SADS‐PL): Initial Reliability and Validity Data.” Journal of the American Academy of Child & Adolescent Psychiatry 36, no. 7: 980–988. 10.1097/00004583-199707000-00021. 9204677

[desc70213-bib-0043] Lawson, G. M. , C. J. Hook , and M. J. Farah . 2018. “A Meta‐Analysis of the Relationship Between Socioeconomic Status and Executive Function Performance Among Children.” Developmental Science 21, no. 2: e12529. 10.1111/desc.12529. PMC5821589 28557154

[desc70213-bib-0044] Lerche, V. , M. von Krause , A. Voss , G. T. Frischkorn , A.‐L. Schubert , and D. Hagemann . 2020. “Diffusion Modeling and Intelligence: Drift Rates Show Both Domain‐General and Domain‐Specific Relations With Intelligence.” Journal of Experimental Psychology: General 149, no. 12: 2207–2249. 10.1037/xge0000774. 32378959

[desc70213-bib-0045] Lerche, V. , and A. Voss . 2019. “Experimental Validation of the Diffusion Model Based on a Slow Response Time Paradigm.” Psychological Research 83, no. 6: 1194–1209. 10.1007/s00426-017-0945-8. 29224184

[desc70213-bib-0046] Lerche, V. , A. Voss , and M. Nagler . 2017. “How Many Trials Are Required for Parameter Estimation in Diffusion Modeling? A Comparison of Different Optimization Criteria.” Behavior Research Methods 49, no. 2: 513–537. 10.3758/s13428-016-0740-2. 27287445

[desc70213-bib-0047] Letkiewicz, A. M. , H. C. Kottler , S. A. Shankman , and A. L. Cochran . 2023. “Quantifying Aberrant Approach‐Avoidance Conflict in Psychopathology: A Review of Computational Approaches.” Neuroscience & Biobehavioral Reviews 147: 105103. 10.1016/j.neubiorev.2023.105103. 36804398 PMC10023482

[desc70213-bib-0048] Levesque, A. R. , S. MacDonald , S. A. Berg , and R. Reka . 2021. “Assessing the Impact of Changes in Household Socioeconomic Status on the Health of Children and Adolescents: A Systematic Review.” Adolescent Research Review 6, no. 2: 91–123. 10.1007/s40894-021-00151-8. 33553578 PMC7853168

[desc70213-bib-0049] Liston, C. , B. S. McEwen , and B. J. Casey . 2009. “Psychosocial Stress Reversibly Disrupts Prefrontal Processing and Attentional Control.” Proceedings of the National Academy of Sciences of the United States of America 106, no. 3: 912–917. 10.1073/pnas.0807041106. 19139412 PMC2621252

[desc70213-bib-0050] Löffler, C. , G. T. Frischkorn , D. Hagemann , K. Sadus , and A.‐L. Schubert . 2024. “The Common Factor of Executive Functions Measures Nothing but Speed of Information Uptake.” Psychological Research 88, no. 4: 1092–1114. 10.1007/s00426-023-01924-7. 38372769 PMC11143038

[desc70213-bib-0051] Lu, S. , L. Perez , A. Leslein , and I. Hatsu . 2019. “The Relationship Between Food Insecurity and Symptoms of Attention‐Deficit Hyperactivity Disorder in Children: A Summary of the Literature.” Nutrients 11, no. 3. 10.3390/nu11030659. PMC6470829 30893802

[desc70213-bib-0052] Mandolesi, L. , F. Gelfo , L. Serra , et al. 2017. “Environmental Factors Promoting Neural Plasticity: Insights from Animal and Human Studies.” Neural Plasticity 2017, no. 1: 7219461. 10.1155/2017/7219461. 28740740 PMC5504954

[desc70213-bib-0053] Mayer, S. E. , and C. Jencks . 1989. “Poverty and the Distribution of Material Hardship.” Journal of Human Resources 24, no. 1: 88–114. 10.2307/145934.

[desc70213-bib-0054] McCarthy, B. , A. Carter , M. Jansson , C. Benoit , and R. Finnigan . 2018. “Poverty, Material Hardship, and Mental Health Among Workers in Three Front‐Line Service Occupations.” Journal of Poverty 22, no. 4: 334–354. 10.1080/10875549.2017.1419532.

[desc70213-bib-0055] McLeod, J. D. , and M. J. Shanahan . 1996. “Trajectories of Poverty and Children's Mental Health.” Journal of Health and Social Behavior 37, no. 3: 207–220. 10.2307/2137292. 8898493

[desc70213-bib-0056] McLoyd, V. C. 1998. “Socioeconomic Disadvantage and Child Development.” American Psychologist 53, no. 2: 185–204. 9491747 10.1037//0003-066x.53.2.185

[desc70213-bib-0057] Mittal, C. , V. Griskevicius , J. A. Simpson , S. Sung , and E. S. Young . 2015. “Cognitive Adaptations to Stressful Environments: When Childhood Adversity Enhances Adult Executive Function.” Journal of Personality and Social Psychology 109, no. 4: 604–621. 10.1037/pspi0000028. 26414842

[desc70213-bib-0058] Moffitt, T. E. , L. Arseneault , D. Belsky , et al. 2011. “A Gradient of Childhood Self‐Control Predicts Health, Wealth, and Public Safety.” Proceedings of the National Academy of Sciences 108, no. 7: 2693–2698. 10.1073/pnas.1010076108. PMC3041102 21262822

[desc70213-bib-0059] Müller, U. , and K. Kerns . 2015. “The Development of Executive Function.” Handbook of Child Psychology and Developmental Science: Cognitive Processes 2: 571–623.

[desc70213-bib-0060] Muthén, B. , and L. Muthén . 2017. “Mplus.” In Handbook of Item Response Theory, edited by W. J. van der Linden , Chapman and Hall/CRC.

[desc70213-bib-0061] Niebaum, J. C. , A. Zengilowski , B. Katz , P. Shah , and Y. Munakata . 2025. “Adaptive Habits: Understanding Executive Function and Its Development.” Trends in Cognitive Sciences 29: 121–134. S1364661325002918. 10.1016/j.tics.2025.10.016. PMC13135620 41412918

[desc70213-bib-0062] Nigg, J. T. 2017. “Annual Research Review: on the Relations Among Self‐regulation, Self‐control, Executive Functioning, Effortful Control, Cognitive Control, Impulsivity, Risk‐Taking, and Inhibition for Developmental Psychopathology.” Journal of Child Psychology and Psychiatry 58, no. 4: 361–383. 10.1111/jcpp.12675. 28035675 PMC5367959

[desc70213-bib-0063] Paige, K. J. , L. M. Cope , J. E. Hardee , et al. 2024. “Leveraging Bifactor Modeling to Test Prospective Direct and Indirect Effects of Adolescent Alcohol Use and Externalizing Symptoms on the Development of Task‐General Executive Functioning.” Development and Psychopathology 37: 1782–1803. 10.1017/S095457942400138X. 39300841 PMC12067474

[desc70213-bib-0064] Paquin, V. , G. Muckle , D. Bolanis , et al. 2021. “Longitudinal Trajectories of Food Insecurity in Childhood and Their Associations With Mental Health and Functioning in Adolescence.” JAMA Network Open 4, no. 12: e2140085. 10.1001/jamanetworkopen.2021.40085. 34928352 PMC8689386

[desc70213-bib-0065] Pechtel, P. , and D. A. Pizzagalli . 2011. “Effects of Early Life Stress on Cognitive and Affective Function: An Integrated Review of human Literature.” Psychopharmacology 214, no. 1: 55–70. 10.1007/s00213-010-2009-2. 20865251 PMC3050094

[desc70213-bib-0098] Perera, F. P. , K. Wheelock , Y. Wang , et al. 2018. “Combined Effects of Prenatal Exposure to Polycyclic Aromatic Hydrocarbons and Material Hardship on Child ADHD Behavior Problems.” Environmental Research 160: 506–513. 10.1016/j.envres.2017.09.002. 28987706 PMC5724364

[desc70213-bib-0066] Petersen, A. C. , L. Crockett , M. Richards , and A. Boxer . 1988. “A Self‐report Measure of Pubertal Status: Reliability, Validity, and Initial Norms.” Journal of Youth and Adolescence 17, no. 2: 117–133. 10.1007/BF01537962. 24277579

[desc70213-bib-0067] Peverill, M. , M. A. Dirks , T. Narvaja , K. L. Herts , J. S. Comer , and K. A. McLaughlin . 2021. “Socioeconomic Status and Child Psychopathology in the United States: A Meta‐Analysis of Population‐Based Studies.” Clinical Psychology Review 83: 101933. 10.1016/j.cpr.2020.101933. 33278703 PMC7855901

[desc70213-bib-0068] Price, R. B. , J. M. Kuckertz , G. J. Siegle , et al. 2015. “Empirical Recommendations for Improving the Stability of the Dot‐Probe Task in Clinical Research.” Psychological Assessment 27, no. 2: 365–376. 10.1037/pas0000036. 25419646 PMC4442069

[desc70213-bib-0069] Rakesh, D. , K. A. McLaughlin , M. Sheridan , K. L. Humphreys , and M. L. Rosen . 2024. “Environmental Contributions to Cognitive Development: The Role of Cognitive Stimulation.” Developmental Review 73: 101135. 10.1016/j.dr.2024.101135. 39830601 PMC11741553

[desc70213-bib-0070] Ratcliff, R. , and G. McKoon . 2008. “The Diffusion Decision Model: Theory and Data for Two‐Choice Decision Tasks.” Neural Computation 20, no. 4: 873–922. 10.1162/neco.2008.12-06-420. 18085991 PMC2474742

[desc70213-bib-0071] Ratcliff, R. , and F. Tuerlinckx . 2002. “Estimating Parameters of the Diffusion Model: Approaches to Dealing With Contaminant Reaction Times and Parameter Variability.” Psychonomic Bulletin & Review 9, no. 3: 438–481. 10.3758/BF03196302. 12412886 PMC2474747

[desc70213-bib-0072] Reichman, N. E. , J. O. Teitler , I. Garfinkel , and S. S. McLanahan . 2001. “Fragile Families: Sample and Design.” Children and Youth Services Review 23, no. 4: 303–326. 10.1016/S0190-7409(01)00141-4.

[desc70213-bib-0073] Rosen, M. L. , M. P. Hagen , L. A. Lurie , et al. 2020. “Cognitive Stimulation as a Mechanism Linking Socioeconomic Status With Executive Function: A Longitudinal Investigation.” Child Development 91, no. 4: e762–e779. 10.1111/cdev.13315. 31591711 PMC7138720

[desc70213-bib-0074] Russell, A. E. , T. Ford , and G. Russell . 2015. “Socioeconomic Associations With ADHD: Findings From a Mediation Analysis.” PLoS ONE 10, no. 6: e0128248. 10.1371/journal.pone.0128248. 26030626 PMC4451079

[desc70213-bib-0075] Russell, A. E. , T. Ford , R. Williams , and G. Russell . 2016. “The Association Between Socioeconomic Disadvantage and Attention Deficit/Hyperactivity Disorder (ADHD): A Systematic Review.” Child Psychiatry & Human Development 47, no. 3: 440–458. 10.1007/s10578-015-0578-3. 26266467

[desc70213-bib-0076] Schenck‐Fontaine, A. , and R. M. Ryan . 2022. “Poverty, Material Hardship, and Children's Outcomes: A Nuanced Understanding of Material Hardship in Childhood.” Children 9, no. 7: 981. 10.3390/children9070981. 35883965 PMC9319381

[desc70213-bib-0077] Schubert, A.‐L. , G. T. Frischkorn , D. Hagemann , and A. Voss . 2016. “Trait Characteristics of Diffusion Model Parameters.” Journal of Intelligence 4, no. 3: 3. 10.3390/jintelligence4030007.

[desc70213-bib-0078] Sheridan, M. A. , F. Shi , A. B. Miller , C. Salhi , and K. A. McLaughlin . 2020. “Network Structure Reveals Clusters of Associations Between Childhood Adversities and Development Outcomes.” Developmental Science 23, no. 5: e12934. 10.1111/desc.12934. 31869484 PMC7308216

[desc70213-bib-0079] Solari, C. D. , and R. D. Mare . 2012. “Housing Crowding Effects on Children's Wellbeing.” Social Science Research 41, no. 2: 464–476. 10.1016/j.ssresearch.2011.09.012. 23017764 PMC3805127

[desc70213-bib-0080] Stafford, T. , A. Pirrone , M. Croucher , and A. Krystalli . 2020. “Quantifying the Benefits of Using Decision Models With Response Time and Accuracy Data.” Behavior Research Methods 52, no. 5: 2142–2155. 10.3758/s13428-020-01372-w. 32232739 PMC7575468

[desc70213-bib-0081] Tomlinson, R. C. , A. S. Weigard , C. Sripada , et al. 2025. “Efficiency of Evidence Accumulation as a Formal Model‐Based Measure of Task‐General Executive Functioning in Adolescents.” Journal of Psychopathology and Clinical Science 134, no. 7: 761–774. 10.1037/abn0001043. 40839478 PMC12373008

[desc70213-bib-0082] Tottenham, N. , J. W. Tanaka , A. C. Leon , et al. 2009. “The NimStim Set of Facial Expressions: Judgments From Untrained Research Participants.” Psychiatry Research 168, no. 3: 242–249. 10.1016/j.psychres.2008.05.006. 19564050 PMC3474329

[desc70213-bib-0083] U. S. Census Bureau . 2023. National Survey of Children's Health, Material Hardship among Children 2022, Data Brief. U.S. Department of Health and Human Services.October 2023.

[desc70213-bib-0084] Vermeent, S. , E. S. Young , M. L. DeJoseph , A.‐L. Schubert , and W. E. Frankenhuis . 2024. “Cognitive Deficits and Enhancements in Youth From Adverse Conditions: An Integrative Assessment Using Drift Diffusion Modeling in the ABCD Study.” Developmental Science 27, no. 4: e13478. 10.1111/desc.13478. 38321588 PMC11338291

[desc70213-bib-0086] Vermeent, S. , E. S. Young , J.‐L. van Gelder , and W. E. Frankenhuis . 2024. “Childhood Adversity Is Not Associated With Lowered Inhibition, but Lower Perceptual Processing: A Drift Diffusion Model Analysis.” Cognitive Development 71: 101479. 10.1016/j.cogdev.2024.101479.

[desc70213-bib-0087] Voss, A. , M. Nagler , and V. Lerche . 2013. “Diffusion Models in Experimental Psychology: A Practical Introduction.” Experimental Psychology 60, no. 6: 385–402. 10.1027/1618-3169/a000218. 23895923

[desc70213-bib-0088] Voss, A. , K. Rothermund , and J. Voss . 2004. “Interpreting the Parameters of the Diffusion Model: An Empirical Validation.” Memory & Cognition 32, no. 7: 1206–1220. 10.3758/BF03196893. 15813501

[desc70213-bib-0089] Vygotsky, L. 1978. “Interaction Between Learning and Development.” In Readings on the Development of Children, edited by M. Gauvain and M. Role , (33–40). Scientific American Books.

[desc70213-bib-0090] Wei, X. , C. J. D. McKinlay , J. E. Harding , et al. 2025. “NIH Toolbox for Assessment of Neurocognitive, Motor and Emotional‐Behavioral Function in Childhood: A Systematic Review.” Child Neuropsychology 31, no. 6: 948–983. 10.1080/09297049.2024.2447444. 40693328

[desc70213-bib-0091] Weigard, A. , and C. Huang‐Pollock . 2017. “The Role of Speed in ADHD‐Related Working Memory Deficits: A Time‐Based Resource‐Sharing and Diffusion Model Account.” Clinical Psychological Science 5, no. 2: 195–211. 10.1177/2167702616668320. 28533945 PMC5437983

[desc70213-bib-0092] Weigard, A. , and C. Sripada . 2021. “Task‐General Efficiency of Evidence Accumulation as a Computationally Defined Neurocognitive Trait: Implications for Clinical Neuroscience.” Biological Psychiatry Global Open Science 1, no. 1: 5–15. 10.1016/j.bpsgos.2021.02.001. 35317408 PMC8936715

[desc70213-bib-0093] Weigard, A. S. , S. J. Brislin , L. M. Cope , et al. 2021. “Evidence Accumulation and Associated Error‐Related Brain Activity as Computationally‐Informed Prospective Predictors of Substance Use in Emerging Adulthood.” Psychopharmacology 238, no. 9: 2629–2644. 10.1007/s00213-021-05885-w. 34173032 PMC8452274

[desc70213-bib-0094] White, C. N. , R. Ratcliff , M. W. Vasey , and G. McKoon . 2010. “Anxiety Enhances Threat Processing Without Competition Among Multiple Inputs: A Diffusion Model Analysis.” Emotion 10, no. 5: 662–677. 10.1037/a0019474. 21038949

[desc70213-bib-0095] Young, E. S. , W. E. Frankenhuis , D. J. DelPriore , and B. J. Ellis . 2022. “Hidden Talents in Context: Cognitive Performance With Abstract Versus Ecological Stimuli Among Adversity‐Exposed Youth.” Child Development 93, no. 5: 1493–1510. 10.1111/cdev.13766. 35404500 PMC9543758

[desc70213-bib-0096] Ziegler, S. , M. L. Pedersen , A. M. Mowinckel , and G. Biele . 2016. “Modelling ADHD: A Review of ADHD Theories Through Their Predictions for Computational Models of Decision‐Making and Reinforcement Learning.” Neuroscience & Biobehavioral Reviews 71: 633–656. 10.1016/j.neubiorev.2016.09.002. 27608958

