# Supplementary material for: Childhood Material Hardship Linked to Adolescent Neurocognition: A Computational Modeling Approach
Source: Dev Sci. 2026 May 5;29:e70213. doi: 10.1111/desc.70213 (PMC13144713; doi:10.1111/desc.70213)
Supplement: Supplementary file 1 — Supporting Material: desc70213‐sup‐0001‐SuppMat.docx [file DESC-29-e70213-s001.docx]

**Supplemental Materials**

**Modeling and Simulation recovery study for DDM**

DDM estimations were conducted with the Dynamic Models of Choice package in R (version 4.3.2, Heathcote et al., 2019; R Core Team, 2023). Beyond the four main DDM parameters (i.e., EEA, response caution, non-decision time, and response bias). Between trial variability of t_0_ was also estimated. Other between trial variability parameters were fixed given the known difficulty of recovering these parameters at low trial numbers (Lerche et al., 2017). Our model also accounted for trial omissions (e.g., due to inattention to task) using a parameter that represents the probability that a response will be omitted (Damaso et al., 2022). This parameter was estimated on the probit scale. Individual level Bayesian estimation was implemented using the differential evolution Markov Chain Monte Carlo (DE-MCMC; Turner et al., 2013) approach with informed Bayesian priors based on a systematic review (Tran et al., 2021). Individual model convergence was assessed by visual examination of the posterior chains and the Gelman-Rubin’s convergence diagnostic (<= 1.1 indicates good convergence; Gelman & Rubin, 1992).

We conducted a simulation-recovery study to assess DDM parameter reliability following procedure from previous studies (Weigard et al., 2023; Weigard et al., 2021). First, parameters estimated from the DDM were used to simulate a new dataset (100 trials per person). The DDM was then fit to the simulated data set using the same procedures outlined above, and parameter estimates were recovered from the simulated data set and compared with the original parameters from the actual performance data. Main text Figure 2 displays scatterplots and correlation *r* between the simulated and recovered parameters, which signals excellent recovery for the key parameters of interest, including drift rate (v), a, t_0_, and z (*r*s > .90). Median values of posterior distributions of DDM parameters were used in the analyses in this study. Main text Table 1 shows the distributions and bivariate correlations between DDM parameters.

**CFA for adolescent attentional problems**

A confirmatory factor analysis was performed to create latent factors of adolescent attentional problems, using item-level responses from the CBCL and K-SADS-PL. The CBCL attention problems subscale included 10 items on child’s behavioral problems related to inattention and hyperactivity in the past 6 months. All items were rated by the caregiver on a scale of 0-2: 0 if the item was not true, 1 if the item was somewhat or sometimes true, and 2 if the item was very true or often true. Adolescent inattention and hyperactivity were also assessed by trained clinical assessors and licensed clinicians with the K-SADS-PL. Assessors administered the semi-structured interview to the adolescent and caregiver individually, arriving at initial DSM-V diagnoses, which were then reviewed in an weekly assessment meeting with the team and licensed clinicians with a final symptoms counts were derived by consensus of the clinical team using best-estimate procedures (Maziade et al., 1992). We used the final clinician rating (on a scale of 0-2, 0 if item behavior not present, 1 if subthreshold, and 2 if met clinical threshold) for each question on inattention and hyperactivity.

We started with a 2-factor model of attentional problems, separating inattention and hyperactivity, based on the theoretical basis that these two were similar but separate constructs (Toplak et al., 2009). The WLSMV estimator was used to account for categorical variables. Each question from the CBCL and K-SADS-PL was loaded onto the two latent factors equally reflect the ADHD symptoms. 1 item from the CBCL was excluded due to low loading based on an a-priori threshold of .40 (“confused or seems to be in a fog”; λ = 0.388). 1 item from the K-SADS-PL was removed (“run/climb excessively”) because of complete overlap with responses of another variable (“easily distracted”). The model showed good fit (CFI = .950, TLI = .946, RMSEA = .044 95% CI [.033, .055], SRMR = .114). However, the latent factors of inattention and hyperactivity were highly correlated (*r* = .86, *p* < .001), and therefore we decided to proceed with a one-factor CFA model of attentional problems with items loading onto one factor. The final model showed good fit (CFI = .922, TLI = .915, RMSEA = .056 95% CI [.046, .065], SRMR = .133). Items and loadings from the final one-factor model is shown in Table S3. Results from the CFA were then extracted into an attentional problems latent factor for analysis in R

**Growth Curve of Material Hardship**

Following the approaches taken in previous studies (Gard et al., 2021; Hardi et al., 2022), linear trajectories were fit on material hardship data over four waves (ages 1, 3, 5, and 9). Material hardship at age 1 was set as a value of 0 as the starting point, and coefficients for ages 3, 5, and 9 are set to 2, 4, and 8, respectively. The model failed to converge with the small subsample included in the analysis. To improve fit and convergence, we utilized the full FFCWS sample data (N = 3714) and extracted the estimated intercepts (initial time point) and slopes (change over time) for the subsample to predict drift rate in a regression analysis in R. The final growth curve model showed excellent fit (CFI = .981, TLI = .977, RMSEA = .042 90% CI [.030, .055], SRMR = .028; (Hu & Bentler, 1999). Material hardship at age 1 was set as point 0, where standardized estimated mean for starting point (i.e., intercept) = 0.98 (*p* < .001, *SE* = .84) and estimated mean change over time (i.e., slope) = .26 (*p* < .001, *SE* = .01).

**Sensitivity Analysis with Violence Exposure**

Analyses reported in the main text adjusted for co-occurring types of adversity, including violence exposure and social deprivation, to estimate the unique association between material hardship and drift rate. At the same time, it is also possible that material hardship may contribute to increased risk of violence exposure (Bywaters et al., 2016; Lacey et al., 2022; Ning et al., 2024) and controlling for it could attenuate the total effect of hardship on drift rate. Therefore, we also tested whether the link between material hardship and drift rate without violence exposure as a covariate. We found that the associations remained significant (cumulative material hardship: 𝛽  = -.18, *p* = .029; growth curve intercept: 𝛽  = -.20, *p* = .014; growth curve slope: 𝛽  = -.07, *p* = .372). We also tested whether violence exposure mediated the effect between material hardship and drift rate. While violence exposure and material hardship are correlated (r =  .43, p < .001), we did not find a significant indirect effect of violence exposure (𝛽  = -.01, b = -.001, 95% CI [-.02, .01]).

**Emotion-specific Drift Rate**

As an exploratory analysis, we estimated the DDM separating trials by emotions on faces. Given the limited number of trials per emotion (n = 20 max), the DDM was set to only vary drift rate by emotions. The model showed good fit and simulation recovery (Figure S1). To find support that drift rate is a task-general trait-like factor, we then extracted a latent factor “emotion-general” drift rate using CFA (conducted in MPlus) from the separate emotions to replicate our previous analyses.

Overall, emotion-specific drift rate estimates were highly correlated with each other, as well as with the “general” drift rate based on all trials in the task, suggesting that drift rate is largely unaffected by the valence of the stimuli in our study (Table S4). We extracted a latent drift rate factor from the emotion-specific drift rate estimates (model fit was excellent: CFI = .993, TLI = .987, RMSEA = .071 90% CI [.000, .137], SRMR = .014). As expected, the latent factor drift rate was highly correlated with the “general” drift rate estimate from the original model (*r* = .99, *p* < .001). We replicated the finding that cumulative childhood material hardship (*β* = -.19, *p* = .026), above and beyond violence exposure (*β* = .01, *p* = .952), social deprivation (*β* = .06, *p* = .518), and demographic covariates, was significantly negatively associated with the latent factor EEA.

We also replicated our findings with attentional problems in adolescents: latent drift rate factor was significantly associated with both inattention (*r* = -.18, *p* = .015) and hyperactivity (*r* = -.18, *p* = .013), and remained significant after adjusting for demographic covariates (inattention: *β* = -.14, *p* = .050; *β* = -.15, *p* = .043).

**Associations of Other DDM Parameters with Adversity and Attentional Problems**

We examined in exploratory analyses whether other parameters from the DDM (i.e., boundary separation (a), non-decision time (t₀), and response bias (z)) were associated with adversity dimensions or attentional problems. Bivariate correlations are reported in Table S5. We found no significant associations between boundary separation or response bias with adversity experiences or attentional problems. However, non-decision time (t₀) was significantly correlated with concurrent attentional problems, such that individuals with greater attentional problems showed lower (i.e., faster) non-decision time (*r* = -.27, *p* < .001). These exploratory findings should be interpreted with caution but highlight the potential value of examining parameters beyond drift rate in future studies.

**Functional Activation and Drift Rate**

We conducted initial exploratory analysis to examine whether the functional activation patterns while participants were performing the emotional-faces gender-identification task were significantly associated with drift rate derived from the behavioral performance data. Past research suggesting that EEA is associated with frontoparietal network activation in response to cognitive load in an N-back task as well as salience network activation in response to errors (Weigard et al., 2024). While we are unable to test for cognitive load and error response directly due to the set-up of the task and our high accuracy rate (93.8%), we conducted an exploratory analysis of whether drift rate is linked with task-engagement by conducting a multiple regression group-level analysis with the contrast condition of faces vs. baseline. fMRI data was collected with a GE Discovery MR750 3T MRI scanner with an 8-channel head coil. Data acquisition, preprocessing, and first-level analysis details are reported in Hein et al. (2018). To example whether drift rate is linked with functional activation, we added drift rate as a covariate in multiple regression for the group-level analysis for the general condition of emotional faces (combined) vs. baseline. Whole-brain analysis was conducted in SPM12 (https://www.fil.ion.ucl.ac.uk/spm/software/spm12/). Results showed no significant clusters at the voxelwise family-wise error rate of p < .050. Similarly, we also conducted group-level analysis for emotion-specific drift rate and emotion-specific contrasts (e.g., fearful drift rate as a covariate, with the fearful vs. baseline contrast). We also did not find any significant clusters. This is likely a reflection of the low cognitive load in our task.

**References**

Bywaters, P., Bunting, L., Davidson, G., Hanratty, J., Mason, W., McCartan, C., & Steils, N. (2016, March 3). *The relationship between poverty, child abuse and neglect: An evidence review*. https://www.jrf.org.uk/child-poverty/the-relationship-between-poverty-child-abuse-and-neglect-an-evidence-review

Damaso, K. A. M., Castro, S. C., Todd, J., Strayer, D. L., Provost, A., Matzke, D., & Heathcote, A. (2022). A cognitive model of response omissions in distraction paradigms. *Memory & Cognition*, *50*(5), 962–978. https://doi.org/10.3758/s13421-021-01265-z

Gelman, A., & Rubin, D. B. (1992). Inference from Iterative Simulation Using Multiple Sequences. *Statistical Science*, *7*(4), 457–472. https://doi.org/10.1214/ss/1177011136

Heathcote, A., Lin, Y.-S., Reynolds, A., Strickland, L., Gretton, M., & Matzke, D. (2019). Dynamic models of choice. *Behavior Research Methods*, *51*(2), 961–985. https://doi.org/10.3758/s13428-018-1067-y

Hein, T. C., Mattson, W. I., Dotterer, H. L., Mitchell, C., Lopez-Duran, N., Thomason, M. E., Peltier, S. J., Welsh, R. C., Hyde, L. W., & Monk, C. S. (2018). Amygdala habituation and uncinate fasciculus connectivity in adolescence: A multi-modal approach. *NeuroImage*, *183*, 617–626. https://doi.org/10.1016/j.neuroimage.2018.08.058

Lacey, R. E., Howe, L. D., Kelly-Irving, M., Bartley, M., & Kelly, Y. (2022). The Clustering of Adverse Childhood Experiences in the Avon Longitudinal Study of Parents and Children: Are Gender and Poverty Important? *Journal of Interpersonal Violence*, *37*(5–6), 2218–2241. https://doi.org/10.1177/0886260520935096

Lerche, V., Voss, A., & Nagler, M. (2017). How many trials are required for parameter estimation in diffusion modeling? A comparison of different optimization criteria. *Behavior Research Methods*, *49*(2), 513–537. https://doi.org/10.3758/s13428-016-0740-2

Maziade, M., Roy, M. A., Fournier, J. P., Cliche, D., Mérette, C., Caron, C., Garneau, Y., Montgrain, N., Shriqui, C., & Dion, C. (1992). Reliability of best-estimate diagnosis in genetic linkage studies of major psychoses: Results from the Quebec pedigree studies. *The American Journal of Psychiatry*, *149*(12), 1674–1686. https://doi.org/10.1176/ajp.149.12.1674

Ning, K., Gondek, D., Pereira, S. M. P., & Lacey, R. E. (2024). Mediating mechanisms of the relationship between exposure to deprivation and threat during childhood and adolescent psychopathology: Evidence from the Millennium Cohort Study. *European Child & Adolescent Psychiatry*, *33*(6), 1907–1920. https://doi.org/10.1007/s00787-023-02289-3

R Core Team. (2023). *R: A language and environment for statistical computing. R Foundation for Statistical Computing*. https://www.R-project.org/

Toplak, M. E., Pitch, A., Flora, D. B., Iwenofu, L., Ghelani, K., Jain, U., & Tannock, R. (2009). The Unity and Diversity of Inattention and Hyperactivity/Impulsivity in ADHD: Evidence for a General Factor with Separable Dimensions. *Journal of Abnormal Child Psychology*, *37*(8), 1137–1150. https://doi.org/10.1007/s10802-009-9336-y

Tran, N.-H., van Maanen, L., Heathcote, A., & Matzke, D. (2021). Systematic Parameter Reviews in Cognitive Modeling: Towards a Robust and Cumulative Characterization of Psychological Processes in the Diffusion Decision Model. *Frontiers in Psychology*, *11*. https://www.frontiersin.org/articles/10.3389/fpsyg.2020.608287

Turner, B. M., Sederberg, P. B., Brown, S. D., & Steyvers, M. (2013). A method for efficiently sampling from distributions with correlated dimensions. *Psychological Methods*, *18*(3), 368–384. https://doi.org/10.1037/a0032222

Weigard, A., Angstadt, M., Taxali, A., Heathcote, A., Heitzeg, M. M., & Sripada, C. (2024). Flexible adaptation of task-positive brain networks predicts efficiency of evidence accumulation. *Communications Biology*, *7*(1), 1–13. https://doi.org/10.1038/s42003-024-06506-w

Weigard, A., McCurry, K. L., Shapiro, Z., Martz, M. E., Angstadt, M., Heitzeg, M. M., Dinov, I. D., & Sripada, C. (2023). Generalizable prediction of childhood ADHD symptoms from neurocognitive testing and youth characteristics. *Translational Psychiatry*, *13*(1), 1–11. https://doi.org/10.1038/s41398-023-02502-6

Weigard, A. S., Brislin, S. J., Cope, L. M., Hardee, J. E., Martz, M. E., Ly, A., Zucker, R. A., Sripada, C., & Heitzeg, M. M. (2021). Evidence accumulation and associated error-related brain activity as computationally-informed prospective predictors of substance use in emerging adulthood. *Psychopharmacology*, *238*(9), 2629–2644. https://doi.org/10.1007/s00213-021-05885-w

| **Table S1. Sample Demographics (n = 187)** | | | | | | |
| --- | --- | --- | --- | --- | --- | --- |
| **Variables** | **Group** | **Mean (SD)** | **Count (Percentage)** | |  |  |
| **Age** | - | 15.83 (*0.51*) | | - | |  |
| **Pubertal Age** | - | 3.26 (*0.58*) | | - | |  |
| **Sex** | Female (0) | - | | 99 (52.94%) | |  |
|  | Male (1) | - | | 88 (47.06%) | |  |
| **Race** | Black | - | | 144 (77.01%) | |  |
|  | White | - | | 22 (11.76%) | |  |
|  | Hispanic/Latino | - | | 11 (5.88%) | |  |
|  | Other/Multiracial | - | | 10 (5.35%) | |  |
| **Family Structure** | Single-parent (0) | - | | 121 (64.71%) | |  |
|  | Two-parent (1) | - | | 66 (35.29%) | |  |
| **Material Hardship** | Cumulative Sum | 4.58 (*4.05*) | | - | |  |
|  | Age 1 | 1.01 (*1.29*) | | - | |  |
|  | Age 3 | 1.06 (*1.31*) | | - | |  |
|  | Age 5 | 1.25 (*1.41*) | | - | |  |
|  | Age 9 | 1.55 (*1.45*) | | - | |  |
| **Violence Exposure** | - | 0 (*0.53*) | |  | |  |
| **Social Deprivation** | - | 0 (*0.50*) | | - | |  |

**Table S2. Zero-order Correlations of Material Hardship Scores**

|  | | *1.* | | *2.* | | *3.* | | *4.* | | *5.* |
| --- | --- | --- | --- | --- | --- | --- | --- | --- | --- | --- |
| *1. Age 1 Hardship* | *-* | |  | |  | |  | |  | |
| *2. Age 3 Hardship* | .54*** | | - | |  | |  | |  | |
| *3. Age 5 Hardship* | .44*** | | .54*** | | - | |  | |  | |
| *4. Age 9 Hardship* | .31*** | | .46*** | | .45*** | | - | |  | |
| *5. Sum Score* | .72*** | | .80*** | | .79*** | | .72*** | | - | |
| ***: p < .001 | | | | | | | | | | |

| **Table S3. Attentional problems one-factor CFA items and loadings** | |
| --- | --- |
| *Item* | *Loading* |
| 1. CBCL – acts too young for his/her age | .60 |
| 2. CBCL – fails to finish things he/she started | .67 |
| 3. CBCL – can’t concentrate, can’t pay attention for long | .82 |
| 4. CBCL – can’t sit still, restless or hyperactive | .78 |
| 5. CBCL – daydreams or gets lost in his/her thoughts | .59 |
| 6. CBCL – impulsive or acts without thinking | .76 |
| 7. CBCL – poor school work | .60 |
| 8. CBCL – inattentive or easily distracted | .90 |
| 9. CBCL – stares blankly | .46 |
| 10. KSADS – careless mistakes | .72 |
| 11. KSADS – can’t pay attention | .88 |
| 12. KSADS – doesn’t listen | .79 |
| 13. KSADS – can’t follow instructions | .75 |
| 14. KSADS – can’t organize | .66 |
| 15. KSADS – avoids attentive tasks | .78 |
| 16. KSADS – loses things | .68 |
| 17. KSADS – easily distracted | .71 |
| 18. KSADS – forgetful | .73 |
| 19. KSADS – often fidgets | .81 |
| 20. KSADS – can’t stay in seat | .85 |
| 21. KSADS – difficulty playing quietly | .87 |
| 22. KSADS – on the go | .67 |
| 23. KSADS – talks excessively | .74 |
| 24. KSADS – blurts out answers | .61 |
| 25. KSADS – difficulty awaiting turn | .86 |
| 26. KSADS – interrupts or intrudes | .78 |
| Note: All items loaded onto the attention problems factors significantly, *p*s < .001 | |

| **Table S4. Bivariate Correlations of Emotion-specific and “General” Drift Rate (v)** | | | | | |  |
| --- | --- | --- | --- | --- | --- | --- |
|  | *1* | *2* | *3* | *4* | *5* | |
| *1. Fearful v* | - | - | - | - | - | |
| *2. Angry v* | .67*** | - | - | - | - | |
| *3. Sad v* | .76*** | .71*** | - | - | - | |
| *4. Neutral v* | .71*** | .65*** | .76*** | - | - | |
| *5. Happy v* | .73*** | .73*** | .72*** | .73*** | - | |
| *6. “General” v* | .87*** | .85*** | .88*** | .87*** | .87*** | |
| ***: p < .001 | | | | | |  |

| **Table S5. Bivariate Correlations of Other DDM Parameters and Childhood Adversity and Attentional Problems Variables** | | | | |
| --- | --- | --- | --- | --- |
|  | *Material Hardship* | *Violence Exposure* | *Social Deprivation* | *Attentional Problems* |
| *Response Caution (a)* | .11 | .06 | .04 | .004 |
| *Non-decision Time (t0)* | -.09 | -.06 | -.07 | -.27*** |
| *Response Bias (z)* | -.02 | .07 | .03 | .08 |
| ****: p < .001* | | | | |


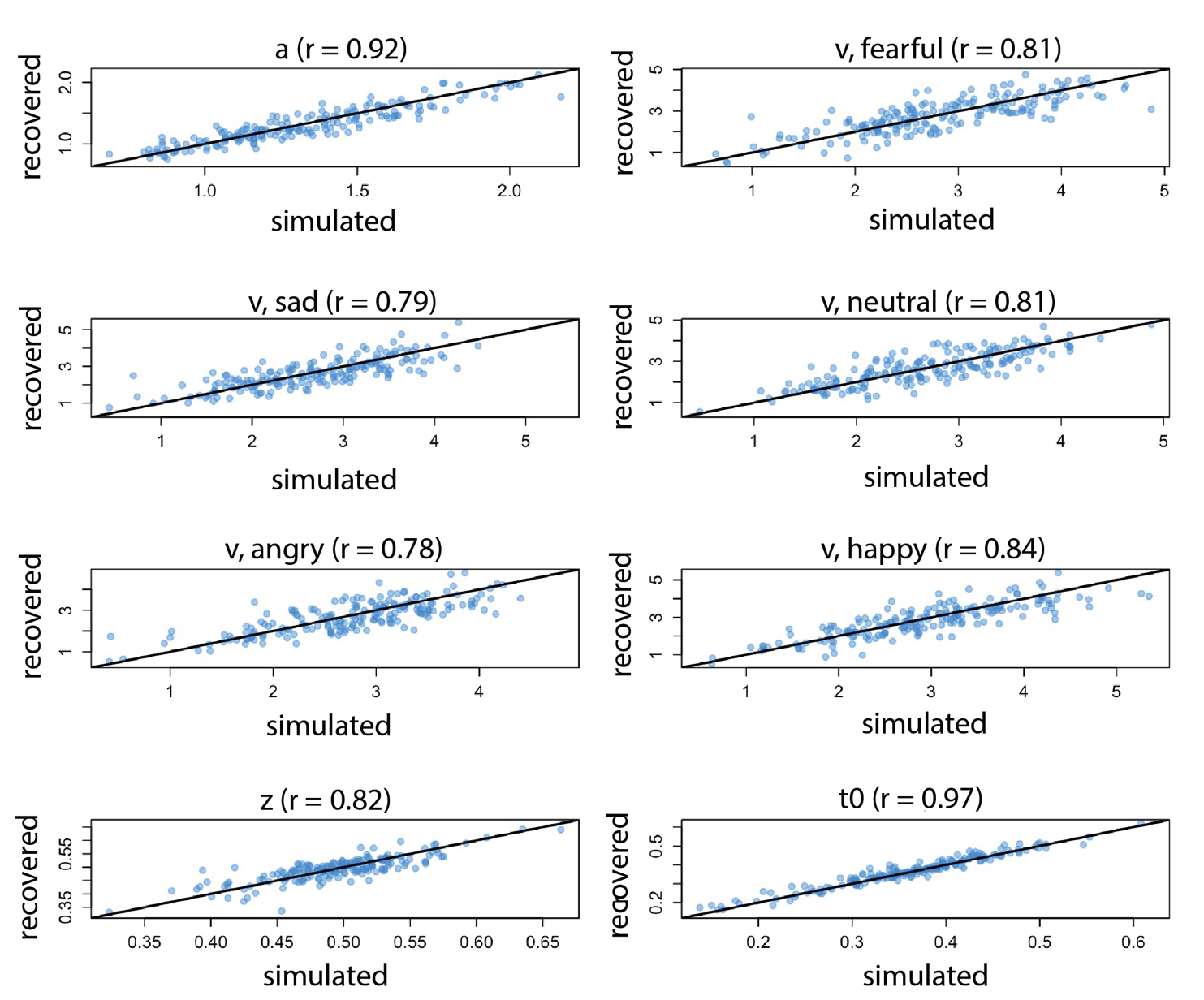


**Figure S1. Simulation-recovery study correlation plots of emotion-specific DDM parameters.** Similar to the previous simulation-recovery study with DDM, parameter values from the DDM (x-axis) was used to simulate another data set, from which another set of parameter values were recovered (y-axis). Correlation values (*r*) between the simulated and recovered parameter values are displayed on each plot. Parameter recovery for emotion-specific drift rate parameters is good (*r*s > .75).
